# Injectable Particulated Human Acellular Dermal Matrix Booster for Skin Restoration: An Integrated Randomized, Split-Face, Double-Blinded Clinical Trial and Preclinical Study

**DOI:** 10.3390/ijms27052193

**Published:** 2026-02-26

**Authors:** Young In Lee, Nam Hao Chau, Ngoc Ha Nguyen, Seoyoon Ham, Yujin Baek, Jihee Kim, Ju Hee Lee

**Affiliations:** 1 Department of Dermatology & Cutaneous Biology Research Institute, Yonsei University College of Medicine, Seoul 03722, Republic of Korea; ylee1124@yuhs.ac (Y.I.L.); nguyenngocha7996@gmail.com (N.H.N.); hsy7852@yuhs.ac (S.H.); uj200076@yuhs.ac (Y.B.); 2 Scar Laser and Plastic Surgery Center, Yonsei Cancer Hospital, Seoul 03722, Republic of Korea; 3 Department of Dermatology, University of Medicine and Pharmacy at Ho Chi Minh City, Ho Chi Minh City 70000, Vietnam; chauhaonamump@gmail.com; 4 Department of Dermatology, Yongin Severance Hospital, Yonsei University College of Medicine, Yongin-si 16995, Republic of Korea; mygirljihee@yuhs.ac

**Keywords:** acellular dermal matrix, dermal fillers, skin boosters, skin aging, extracellular matrix, regenerative medicine

## Abstract

Injectable skin boosters currently in use mainly provide short-lived volumization or depend on inflammation-mediated collagen stimulation, raising concerns regarding durability and safety. Injectable particulate human acellular dermal matrix (phADM) is a biologically derived extracellular matrix scaffold designed to support constructive dermis remodeling. This randomized, split-face, double-blinded clinical trial evaluated the efficacy of phADM as a facial skin booster in 20 adults with moderate cheek roughness. phADM was injected on one facial side, with hyaluronic acid serving as the contralateral control. Multiple skin parameters were assessed over 20 weeks using validated imaging and biophysical instruments. Mechanistic validation was conducted using complementary in vitro, ex vivo human skin, and in vivo rat models. Clinically, the phADM-treated side demonstrated greater improvements in skin density, volume, elasticity, wrinkle depth, pore area, hydration, and barrier-related parameters at multiple time points compared with HA. In ex vivo human skin, phADM showed homogeneous dermal distribution and preservation of extracellular matrix architecture, along with restoration of basement membrane-associated proteins following UVB irradiation. In vivo rat studies revealed fibroblast infiltration and localized neocollagenesis within the implanted matrix. In vitro assays further indicated enhanced fibroblast proliferation and extracellular matrix synthesis, increased hyaluronan production, suppression of pro-inflammatory cytokines in activated macrophages, and downregulation of melanogenesis-related genes in melanoma cells. No serious adverse events were observed during the clinical study. These findings indicate that phADM functions as a restorative skin booster that promotes durable dermis remodeling and functional rejuvenation with a favorable safety profile.

## 1. Introduction

Currently, the landscape of non-surgical facial rejuvenation is dominated by skin boosters, defined as bioactive materials injected into the skin to promote its functions, and restore and maintain its structure. Most commonly used skin boosters include hyaluronic acid (HA), polynucleotides, and synthetic collagen stimulators [1,2]. HA fillers primarily offer volumetric augmentation and hydration with low biostimulation, while synthetic stimulators [e.g., poly-L-lactic acid (PLLA), calcium hydroxyapatite (CaHA)] induce prolonged collagen production through an inflammatory foreign body reaction [3,4,5,6,7]. However, neither approach fully recapitulates the natural skin environment. Moreover, these synthetic stimulators are associated with inherent safety concerns, including the risk of foreign body granulomas and delayed-type hypersensitivity reactions [8,9,10,11]. These limitations highlight the unmet need for next-generation skin boosters that deliver fundamental skin improvement through biocompatible regenerative mechanisms while avoiding the safety liabilities associated with synthetic materials.

In this context, human acellular dermal matrix (hADM) represents an important advance in restorative medicine as a biologically derived scaffold that preserves native extracellular matrix (ECM) architecture while removing cellular components to reduce immunogenicity and the risk of immune rejection [12]. As a homologous biomaterial derived from human tissue, hADM retains the ultrastructural organization of the dermis, including collagen, elastin, and glycosaminoglycans, thereby conferring mechanical stiffness and elastic properties that closely approximate those of native human skin [13,14]. Upon implantation into the skin, hADM promotes vascularization, host cell infiltration, and incorporation into the tissues within 3–6 months, thereby enhancing production of ECM components (e.g., collagen and elastin) while preserving its long-term structural integrity [13,15]. While hADM has traditionally been used in sheet forms for reconstructive surgery [16,17,18,19], its evolution into a micronized/particulated, injectable form has opened new frontiers in aesthetic dermatology [17,20,21]. However, clinical studies investigating injectable hADM as a primary dermal scaffold for tissue restoration and homeostatic skin remodeling are still absent.

To address this knowledge gap, we designed this study as a randomized, split-face, double-blinded, prospective clinical trial to evaluate the effectiveness and safety of phADM when utilized as a skin booster. Furthermore, to substantiate the clinical outcomes and clarify underlying mechanisms, we conducted integrated in vitro, ex vivo, and in vivo studies examining dermis structural remodeling, skin barrier restoration, and regulation of inflammatory and pigmentation pathways. Collectively, these investigations provide a scientific basis for evaluating the therapeutic potential of phADM in skin restoration.

## 2. Results

### 2.1. Participants’ Characteristics

**Figure 1:**
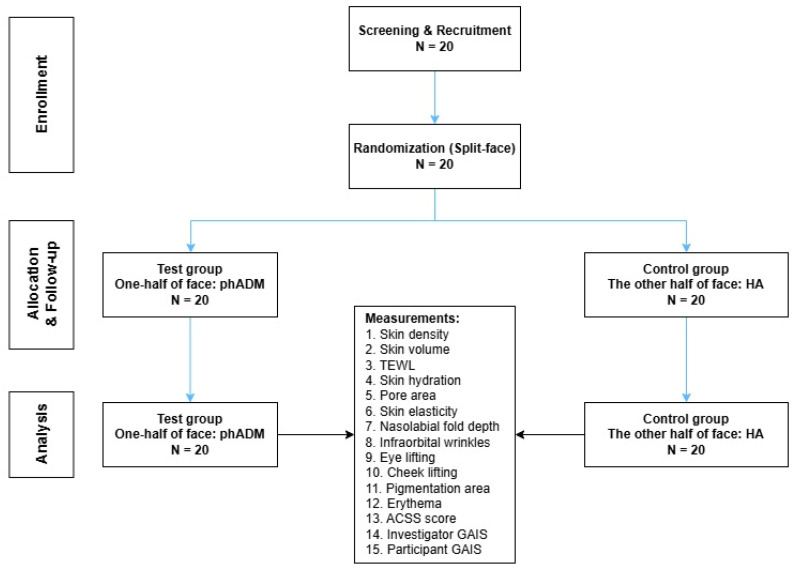
Flowchart of the Screening, Recruitment, Randomization, and Follow-up process of the study. phADM, particulated human Acellular Dermal Matrix; HA, hyaluronic acid; TEWL, transepidermal water loss; ACSS, Allergan Cheek Smoothness Scale; GAIS, Global Aesthetic Improvement Scale.

Overall, 20 subjects were recruited for the study. The mean age was 54.7 ± 9.246 years, with 13 of the 20 participants being female (Table 1). There were no significant differences in Allergan Cheek Smoothness Scale (ACSS) scores between the control and test sides of participants’ faces (*p* = 0.999). The process of screening, recruitment, randomization, and follow-up is depicted in Figure 1.

### 2.2. Structural Restoration: Rebuilding the Dermis Architecture via phADM

#### 2.2.1. Increased Skin Density and Volumetric Enhancement via Structural Support

**Figure 2:**
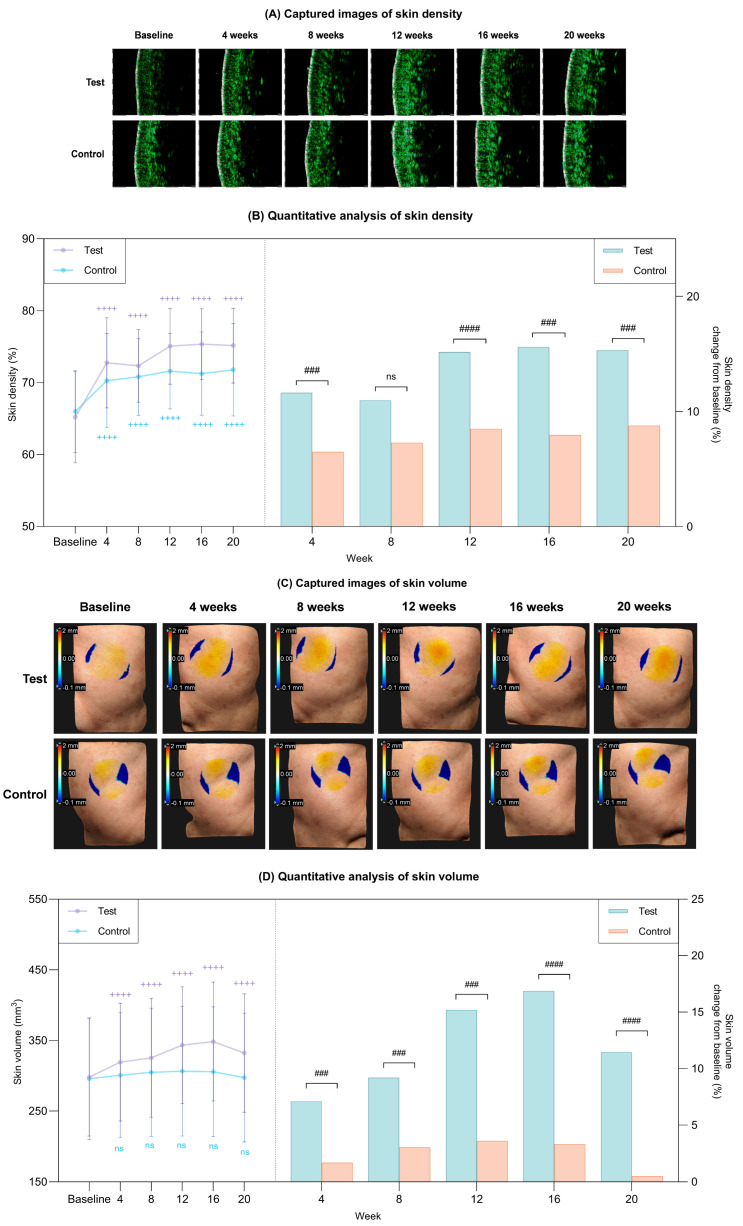
Clinical improvements in skin density and volume after 20 weeks of treatment in the test and control groups. (**A**) Captured images of skin density via Skin Scanner. (**B**) Quantitative measurement of skin density (left) and percent change from baseline (right) in each group. (**C**) Captured images of skin volume via 3D LifeViz micro. (**D**) Quantitative measurement of skin volume (left) and percent change from baseline (right) in each group. Data are presented as mean ± SD. + comparison to baseline within groups, ++++ *p* < 0.001. # comparison of change from baseline between groups at each time point, ### *p* < 0.005, #### *p* < 0.001. ns, not significant.

The longitudinal changes in skin density for both treatment groups are visualized in Figure 2A. Quantitatively, while both groups demonstrated statistically significant improvements from baseline at week 20, the magnitude of improvement in the test group was significantly greater (*p* < 0.05; Figure 2B, Table 2). Figure 2C illustrates the changes in skin volume for both groups over the 20-week period. Quantitative analysis revealed that the test group showed a marked increase from baseline after 20 weeks, whereas the control group exhibited minimal changes (Figure 2D). Consequently, the test group demonstrated superior volumetric enhancement compared to the control group at all-time points (*p* < 0.05; Figure 2D, Table 2).

#### 2.2.2. Histological and Architectural Remodeling

**Figure 3:**
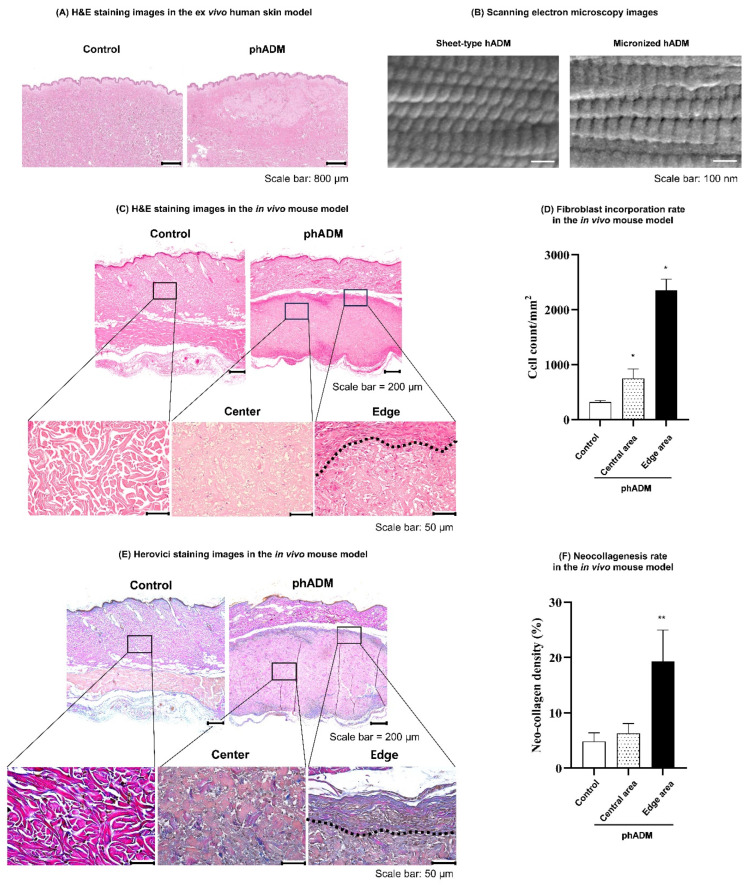
(**A**) Histological images of ex vivo human skin tissues 24 h after injection of phADM or HA. (**B**) Scanning electron microscopy (SEM) analysis of collagen microarchitecture in sheet-type and phADM (magnification: 50,000×). (**C**–**F**) Histological assessment in the in vivo mouse model 1 week after intradermal injection of phADM. (**C**) H&E staining images in the control and phADM samples. (**D**) Fibroblast incorporation rate in the control group, the central and edge areas of the phADM group. (**E**) Herovici staining images in the control and phADM samples. Newly formed collagen are the blue-stained regions. (**F**) Fibroblast incorporation rate in the control group, the central and edge areas of the phADM group. Data are presented as mean ± SD. * comparison to the control group, * *p* < 0.05, ** *p* < 0.01. H&E, hematoxylin and eosin. phADM, particulated human acellular dermal matrix.

Histological evaluation of ex vivo human skin 24 h post-intradermal injection revealed that phADM displayed a homogeneous and diffuse distribution within the dermis, preserving the native tissue architecture (Figure 3A).

High-magnification scanning electron microscopy (SEM) analysis demonstrated that the ECM architecture of phADM was well preserved (Figure 3B). Compared with sheet-type hADM, phADM retained comparable ultrastructural features following micronization, including well-aligned collagen fibrils and a characteristic D-banding pattern arranged in an orderly manner. These findings indicate that the native collagen ultrastructure and overall decellularized ECM architecture remained intact throughout the particulation and manufacturing processes.

In the in vivo rat model, pronounced fibroblast migration and integration into the implanted matrix were observed one week after intradermal injection of phADM (Figure 3C). This was accompanied by a significantly increased fibroblast density compared to controls (Figure 3D). Fibroblast accumulation was evident in both the central and peripheral regions of the phADM, with the highest density noted at the matrix–host interface. Consistently, Herovici (HV) staining demonstrated enhanced neocollagenesis in phADM-treated skin, predominantly at the matrix–host interface (Figure 3E). Quantitative analysis confirmed that neocollagen density was significantly higher in the peripheral region than in both the control and central regions (Figure 3F), suggesting localized collagen synthesis coupled with fibroblast migration and matrix integration.

### 2.3. Functional Improvement of the Skin via hADM: Enhancing Skin Barrier

#### 2.3.1. Changes in Transepidermal Water Loss (TEWL) and Skin Hydration

**Figure 4:**
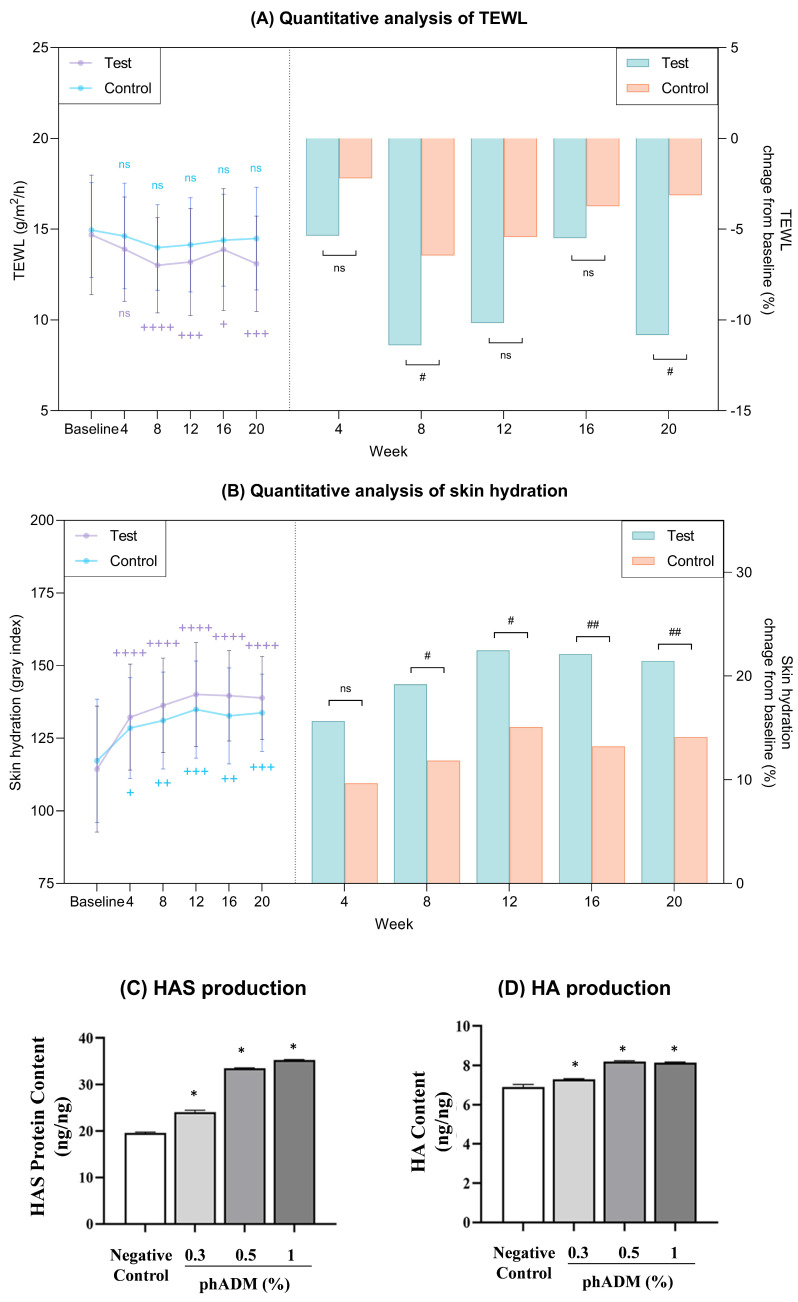
(**A**,**B**) Clinical reduction in TEWL and improvement in skin hydration after 20 weeks in the test and control groups. (**A**) Quantitative measurement of TEWL (left) and percent change from baseline (right) in each group. (**B**) Quantitative measurement of skin hydration (left) and percent change from baseline (right) in each group. (**C**,**D**) phADM’s effect on hyaluronic acid synthesis in human epidermal keratinocytes. The production of HAS (**C**) and HA (**D**) significantly increased 24 h after phADM treatment with increasing concentrations (0.3–1%). Data are presented as mean ± SD. + comparison to baseline within groups, + *p* < 0.05, ++ *p* < 0.01, +++ *p* < 0.005, ++++ *p* < 0.001. # comparison of change from baseline between groups at each time point, # *p* < 0.05, ## *p* < 0.01. TEWL, transepidermal water loss; ns, not significant. ∗ comparison with negative control, ∗ *p* < 0.05. HA, hyaluronic acid. HAS, hyaluronic acid synthase. phADM, particulated human acellular dermal matrix.

Throughout the 20-week study, the test group demonstrated a marked reduction in TEWL (*p* < 0.05), in contrast to the changes observed in the control group (Figure 4A, Table 2). Comparative analysis revealed that the test group achieved significantly superior TEWL improvement relative to the control group at weeks 8 and 20 (*p* < 0.05), underscoring the early and long-term efficacy of phADM in enhancing skin barrier integrity. Regarding skin hydration, while both groups showed progressive improvement, the test group exhibited significantly superior hydration levels compared to controls from week 8 onwards (*p* < 0.05; Figure 4B, Table 2).

For the in vitro validation setting, human epidermal keratinocytes (HEKs) were exposed to varying doses of phADM for 24 h and exhibited no cytotoxic effects compared to negative controls (Figure S2). Based on these data, concentrations of 0.3%, 0.5%, and 1% were utilized for further assays. The evaluation of HA synthesis revealed that phADM treatment for 24 h significantly increased hyaluronan synthase (HAS) protein levels in a dose-dependent manner (*p* < 0.05; Figure 4C). Moreover, the intracellular HA content was significantly elevated, particularly at 0.5% and 1% concentrations (*p* < 0.05; Figure 4D).

#### 2.3.2. Upregulation of Basement Membrane Components in an Ex Vivo Photoaging Model

**Figure 5:**
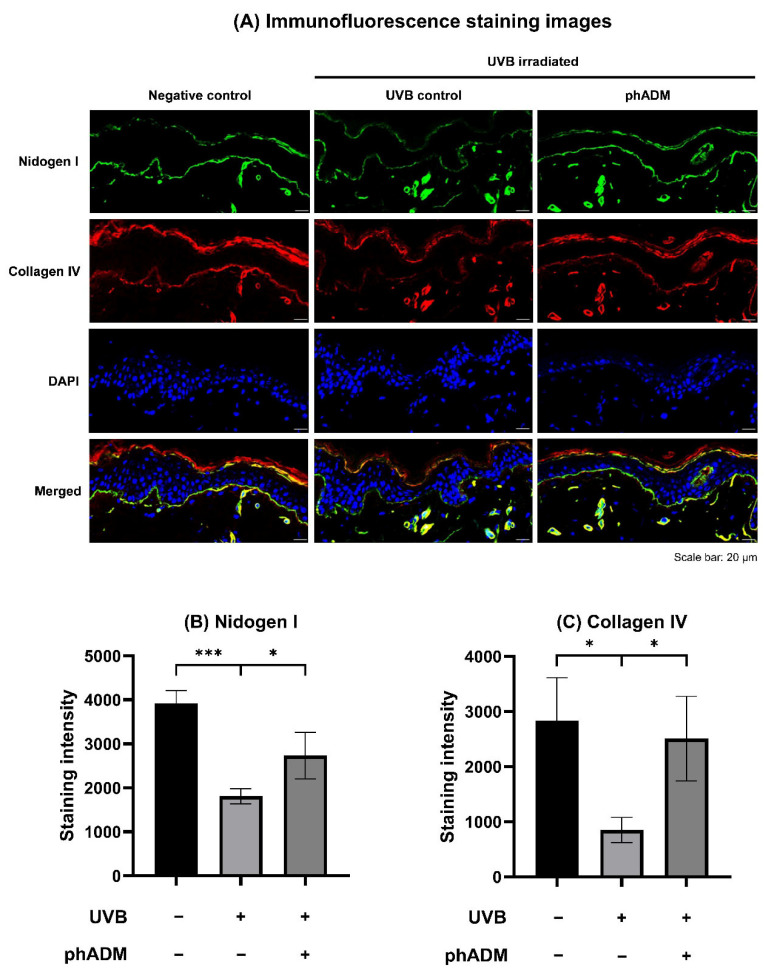
Immunofluorescence assessment in the UVB-irradiated ex vivo human skin model. (**A**) Immunofluorescence staining images. Nidogen I is stained green, Collagen IV is stained red, and DAPI is stained blue. Scale bar = 20 µm. Quantitative analysis of the staining intensity of Nidogen I (**B**) and Collagen IV (**C**). Data are presented as mean ± SD. ∗ comparison between groups, ∗ *p* < 0.05, ∗∗∗ *p* < 0.005. DAPI, 4′,6-diamidino-2-phenylindole. phADM, particulated human acellular dermal matrix. UVB, ultraviolet B.

To validate the restorative effects of phADM on the basement membrane, we utilized an ultraviolet B (UVB)-irradiated ex vivo human skin model to assess the expression of key structural proteins. Immunofluorescence analysis conducted 72 h post-injection revealed significant restoration of basement membrane integrity. Specifically, while UVB irradiation markedly disrupted and downregulated nidogen I and collagen IV staining along the dermal–epidermal junction (DEJ), phADM treatment preserved the continuity and intensity of both markers (Figure 5A). Quantitative analysis confirmed a significant recovery of nidogen I and collagen IV expression in phADM-treated skin compared to UVB-irradiated controls (*p* < 0.05; Figure 5B,C), suggesting that phADM facilitates rapid basement membrane repair following UVB-induced damage.

### 2.4. Restoration of the Skin Function at the Molecular Level

#### 2.4.1. Enhanced Proliferation of Human Dermal Fibroblasts (HDFs) After phADM Treatment

**Figure 6:**
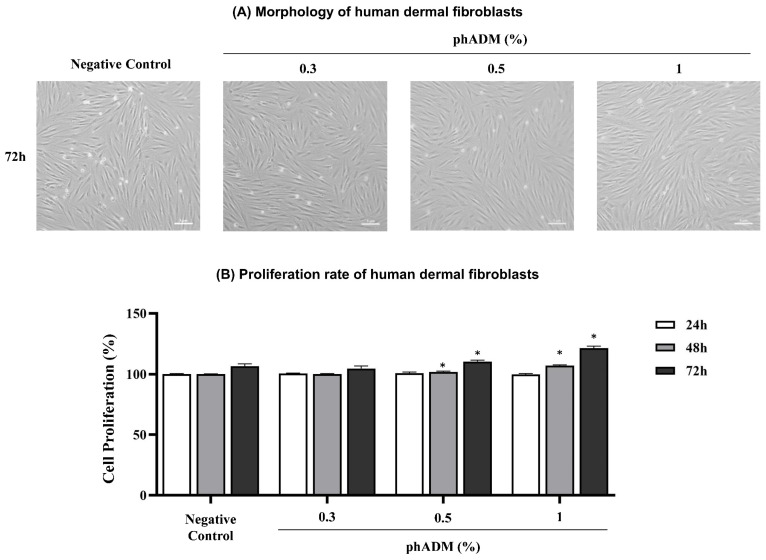
(**A**) Morphology of human dermal fibroblasts after 72 h of treatment with increasing concentrations of phADM (0.3–1%), scale bar: 5 μm. (**B**) Proliferation rate of human dermal fibroblasts after 24, 48, and 72 h of treatment with increasing concentrations of phADM (0.3–1%). Data are presented as mean ± SD. ∗ comparison to the negative control at the same time point, ∗ *p* < 0.05. phADM, particulated human acellular dermal matrix.

The effects of phADM on HDFs proliferation were evaluated following treatment with increasing concentrations of phADM (0.3%, 0.5%, and 1%) for 24, 48, and 72 h. Morphological assessment showed that HDFs maintained a typical spindle-shaped phenotype across all phADM concentrations, with no evidence of cytotoxicity or abnormal cellular morphology at 72 h (Figure 6A).

Quantitative analysis demonstrated a concentration- and time-dependent increase in HDF proliferation after the phADM treatment (*p* < 0.05, Figure 6B). While no significant differences were observed at 24 h, treatment with 0.5% and 1% phADM significantly enhanced cell proliferation at 48 and 72 h compared with the negative control.

#### 2.4.2. Activation of ECM Synthesis and Pro-Regenerative Growth Factors

**Figure 7:**
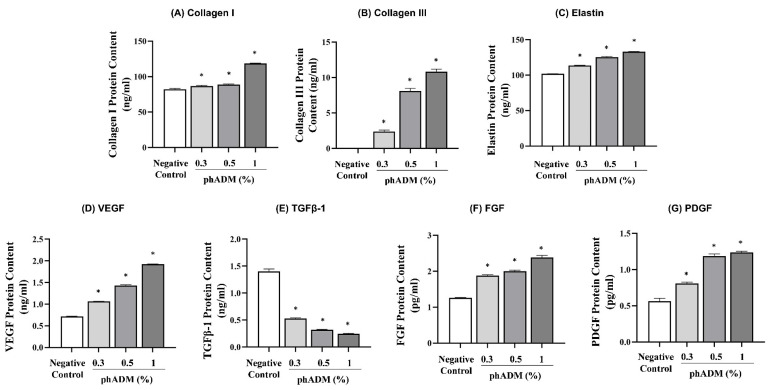
phADM’s effect on the production of collagen, elastin, and growth factors in human dermal fibroblasts. The production of (**A**) Collagen I, (**B**) Collagen III, (**C**) Elastin, (**D**) VEGF, (**E**) TGF-1, (**F**) FGF, and (**G**) PDGF significantly increased 24 h after phADM treatment with increasing concentrations (0.3–1%). Data are presented as mean ± SD. ∗ comparison with negative control, ∗ *p* < 0.05. VEGF, vascular endothelial growth factor. TGFβ-1, transforming growth factor beta 1. FGF, fibroblast growth factor. PDGF, platelet-derived growth factor. phADM, particulated human acellular dermal matrix.

The effects of phADM on extracellular matrix (ECM) protein synthesis and growth factor production were evaluated in HDFs 24 h after treatment with increasing concentrations of phADM (0.3–1%). phADM significantly increased the protein levels of major structural ECM components, including collagen I, collagen III, and elastin, in a concentration-dependent manner (*p* < 0.05, Figure 7A–C). In parallel, phADM markedly enhanced the production of pro-regenerative growth factors, such as vascular endothelial growth factor (VEGF), fibroblast growth factor (FGF), platelet-derived growth factor (PDGF), while significantly reducing transforming growth factor-β1 (TGF-β1) levels compared with the negative control (*p* < 0.05, Figure 7D–G). These results indicate that phADM promotes a regenerative ECM remodeling profile in HDFs by stimulating matrix synthesis and angiogenic growth factor production while limiting pro-fibrotic signaling.

#### 2.4.3. Anti-Inflammatory Effects of phADM in Activated Macrophages

**Figure 8:**
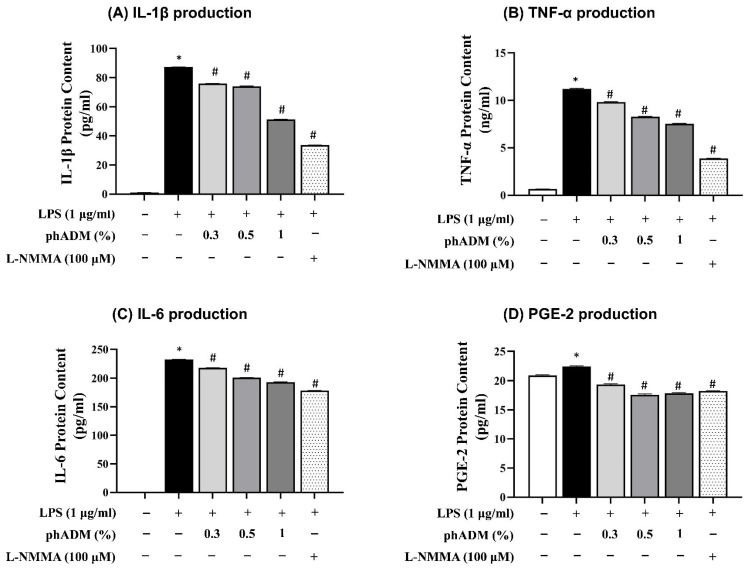
Anti-inflammatory properties of phADM in mouse macrophages. After 24 h of treatment with LPS (1 µg/mL), the production of IL-1β (**A**), TNF-α (**B**), IL-6 (**C**), and PGE-2 (**D**) in mouse macrophages significantly increased. Treatment with phADM (0.3–1%) successfully counteracted this increase. L-NMMA (100 µM) served as positive control. Data are presented as mean ± SD. ∗ comparison with negative control, ∗ *p* < 0.05. # comparison with LPS control, # *p* < 0.05. LPS, lipopolysaccharide. phADM, particulated human acellular dermal matrix. L-NMMA, N-monomethyl-L-arginine. IL, interleukin. TNF-α, tumor necrosis factor–alpha. PGE-2, prostaglandin E2.

Following 24 h of exposure to increasing concentrations of phADM, mouse macrophages exhibited no significant changes in cell viability compared with the negative control (Figure S1). Based on these results, phADM concentrations of 0.3, 0.5, and 1% were selected for subsequent experiments. To evaluate the anti-inflammatory effects of phADM, mouse macrophages were stimulated with lipopolysaccharide (LPS, 1 µg/mL) and co-treated with phADM (0.3–1%) for 24 h, with NG-methyl-L-arginine acetate salt (L-NMMA, 100 µM) used as a positive control. LPS stimulation markedly increased the production of pro-inflammatory mediators, including interleukin-1β (IL-1β), interleukin-6 (IL-6), tumor necrosis factor-α (TNF-α), and prostaglandin E_2_ (PGE-2). Treatment with phADM significantly and dose-dependently suppressed LPS-induced cytokine and mediator production across all measured endpoints (*p* < 0.05; Figure 8A–D). Collectively, these findings demonstrate that phADM exerts potent anti-inflammatory effects in activated macrophages.

### 2.5. Clinical Manifestations of ECM-Driven Skin Rejuvenation

#### 2.5.1. Subjective Assessments: ACSS and GAIS Scores

**Figure 9:**
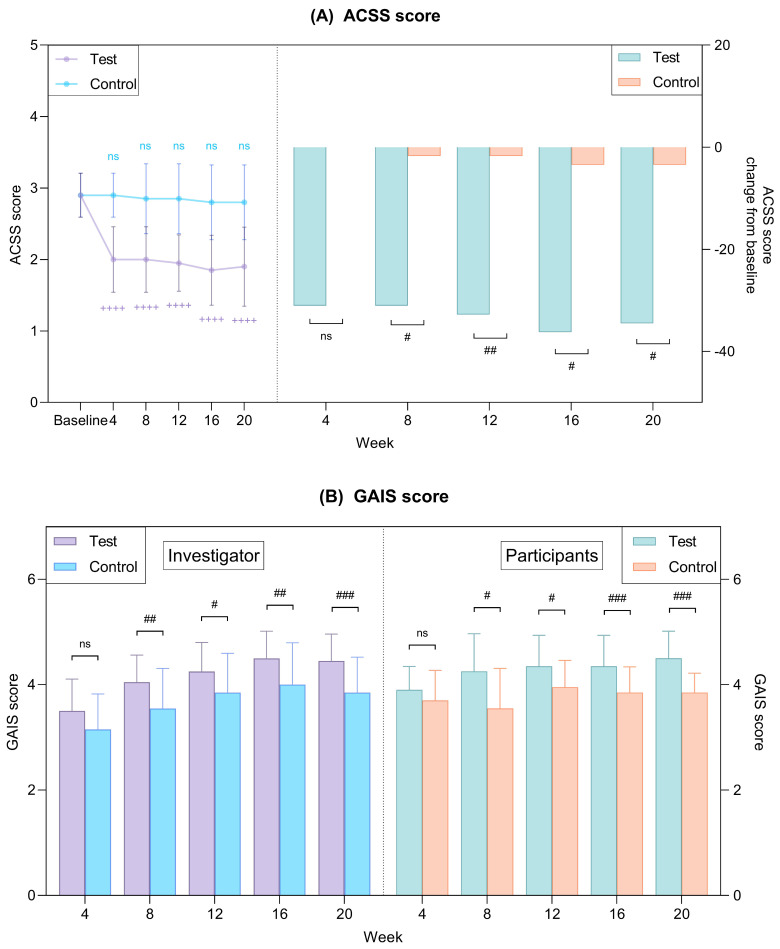
Subjective evaluation from the investigators and participants. (**A**) ACSS scores. (**B**) GAIS scores from the investigators and participants. Data are presented as mean ± SD. + comparison to baseline within groups, ++++ *p* < 0.001. # comparison between groups at each time point, # *p* < 0.05, ## *p* < 0.01, ### *p* < 0.005. ns, not significant. ACSS, Allergan Cheek Smoothness Scale. GAIS, Global Aesthetic Improvement Scale.

Regarding ACSS scores, the test group demonstrated a statistically significant and sustained reduction from baseline at all assessment time points (*p* < 0.05; Figure 9A, Table 2), whereas the control group showed no meaningful change throughout the 20-week study period. Consequently, the magnitude of the ACSS score reduction was significantly greater in the test group than in the control group.

Investigator-assessed Global Aesthetic Improvement Scale (GAIS) scores in the test group were significantly higher than those in the control group from week 8 through week 20 (*p* < 0.05). Consistent with these findings, participant-assessed GAIS scores also exhibited a similar pattern, with significantly greater improvements observed in the test group compared with the control group (*p* < 0.05; Figure 9B, Table 2).

#### 2.5.2. Wrinkle Reduction and Lifting Effect

**Figure 10:**
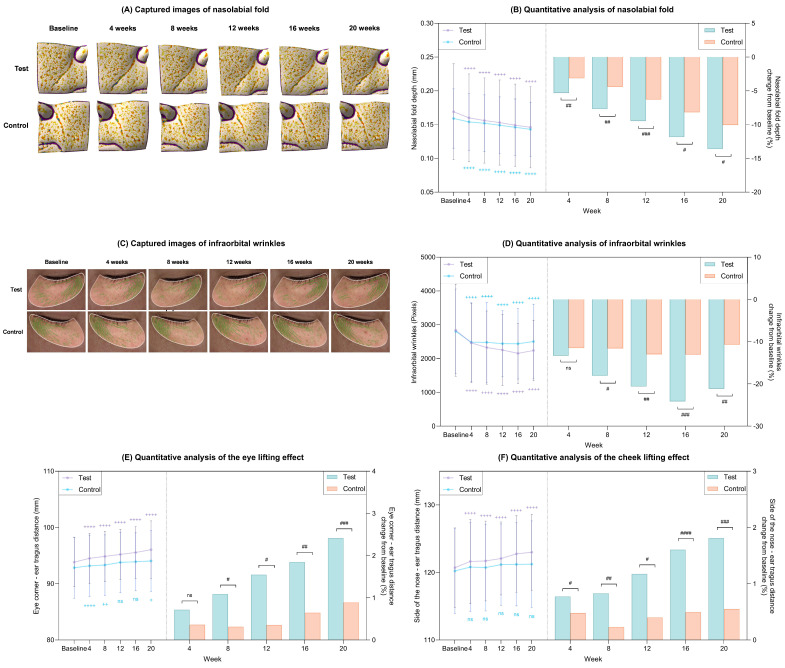
Clinical wrinkle reductions and lifting effects after 20 weeks of treatment in the test and control groups. (**A**) Captured images of nasolabial folds via Antera 3D CS. (**B**) Quantitative measurement of nasolabial fold depth (left) and percent change from baseline (right) in each group. (**C**) Captured images of infraorbital wrinkles via Eve V. (**D**) Quantitative measurement of infraorbital wrinkles (left) and percent change from baseline (right) in each group. (**E**) Quantitative measurement of the contour of the eye area (left) and percent change from baseline (right) in each group. (**F**) Quantitative measurement of the contour of the cheek area (left) and percent change from baseline (right) in each group. Data are presented as mean ± SD. + comparison to baseline within groups, + *p* < 0.05, ++ *p* < 0.01, ++++ *p* < 0.001. # comparison of change from baseline between groups at each time point, # *p* < 0.05, ## *p* < 0.01, ### *p* < 0.005, #### *p* < 0.001. ns, not significant.

Representative clinical images illustrate visible reductions in nasolabial fold and infraorbital wrinkles in both groups after 20 weeks of treatment (Figure 10A,C). Quantitative analyses revealed that nasolabial fold depth (Figure 10B) and infraorbital wrinkle severity (Figure 10D) were significantly reduced from baseline at all evaluated time points over the 20-week period in the test group (*p* < 0.05; Table 2). Moreover, these improvements were significantly greater in the test group than in the control group at corresponding visits (*p* < 0.05).

Assessment of lifting effects at week 20 demonstrated that the test group achieved significantly more pronounced lifting of both the periorbital and cheek regions compared with baseline and with the control group (*p* < 0.05; Figure 10E,F).

#### 2.5.3. Pore Area Reduction and Restoration of Skin Elasticity

**Figure 11:**
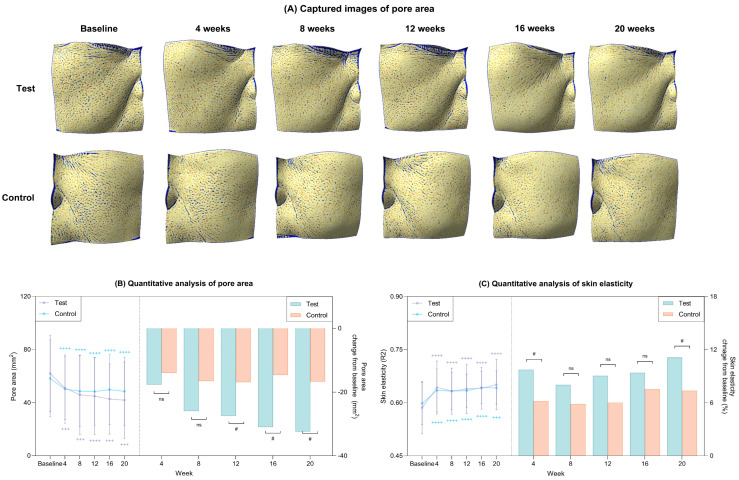
Clinical reductions in pore area and improvement in skin hydration after 20 weeks of treatment in the test and control groups. (**A**) Captured images of pores via Antera 3D CS. (**B**) Quantitative measurement of pore area (left) and percent change from baseline (right) in each group. (**C**) Quantitative measurement of skin elasticity (left) and percent change from baseline (right) in each group. Data are presented as mean ± SD. + comparison to baseline within groups, +++ *p* < 0.005, ++++ *p* < 0.001. # comparison of change from baseline between groups at each time point, # *p* < 0.05. ns, not significant.

Changes in pore area over the 20-week study period are illustrated in Figure 11A. Quantitative analyses demonstrated a progressive and statistically significant reduction in pore area from baseline in both groups throughout the trial (*p* < 0.05; Figure 11B, Table 2). Notably, the magnitude of pore area reduction was significantly greater in the test group than in the control group from week 12 onward (*p* < 0.05; Figure 11B, Table 2), indicating a sustained treatment effect of phADM on pore area reduction over time. With respect to skin elasticity, both groups exhibited significant improvements from baseline at week 20 (*p* < 0.05; Figure 11C, Table 2). However, the test group showed significantly greater enhancement in skin elasticity at weeks 4 and 20 compared with the control group (*p* < 0.05; Figure 11C, Table 2).

#### 2.5.4. Improvement of Age-Related Pigmentation

**Figure 12:**
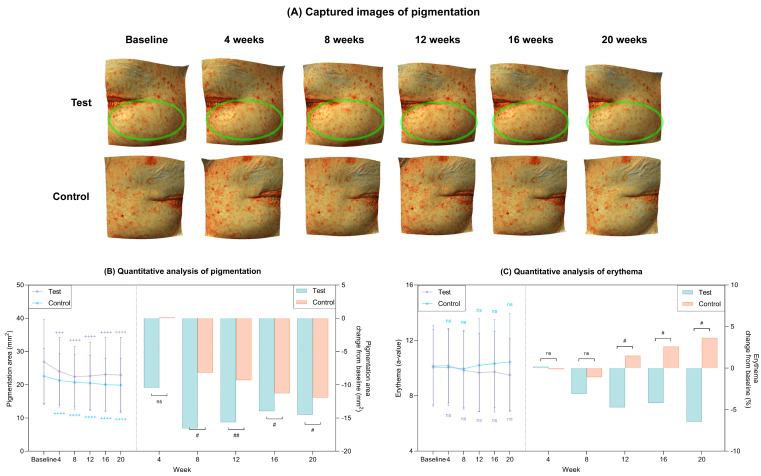
Clinical reductions in pigmentation area and erythema after 20 weeks of treatment in the test and control groups. (**A**) Captured images of pigmentation via Antera 3D CS. The green eclipses indicate the areas of improvement. (**B**) Quantitative measurement of pigmentation area (left) and percent change from baseline (right) in each group. (**C**) Quantitative measurement of erythema (left) and percent change from baseline (right) in each group. Data are presented as mean ± SD. + comparison to baseline within groups, +++ *p* < 0.005, ++++ *p* < 0.001. # comparison of change from baseline between groups at each time point, # *p* < 0.05, ## *p* < 0.01. ns, not significant.

Representative Antera 3D images illustrate clinical changes in pigmentation area after 20 weeks in the test and control groups (Figure 12A). Quantitative analysis revealed a significant and progressive reduction in pigmentation area in the test group over the 20-week study period (*p* < 0.05; Figure 12B, Table 2), with reductions that were significantly greater than those observed in the control group (*p* < 0.05). Meanwhile, the erythema index decreased in the test group, whereas an increasing trend was observed in the control group (Figure 12C, Table 2).

To further substantiate the depigmenting effects of phADM observed in the clinical setting, mechanistic validation studies were conducted using in vitro mouse melanoma cells. Following 72 h of treatment with increasing concentrations of phADM, melanoma cell viability remained comparable to that of the negative control across all tested conditions (Figure S3). Based on these findings, phADM concentrations of 0.3, 0.5, and 1% were selected for subsequent experiments.

**Figure 13:**
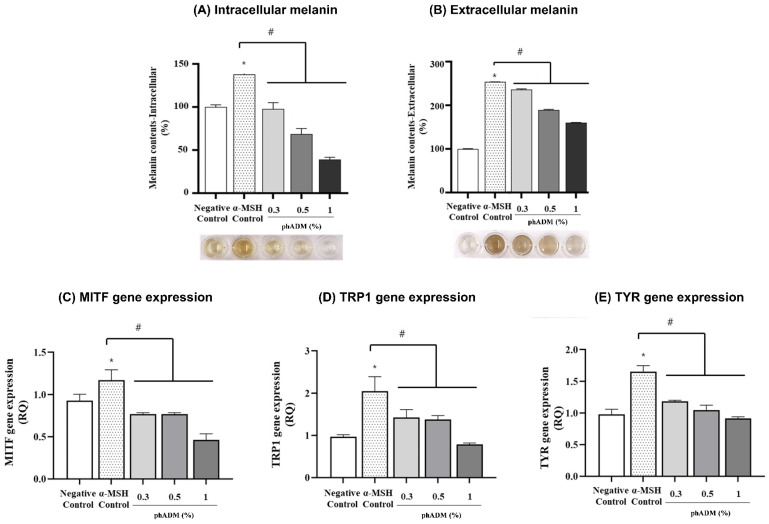
Depigmentation effect of phADM in mouse melanoma cells. After 72 h, α-MSH treatment significantly increased the production of intracellular melanin (**A**) and extracellular melanin (**B**), as well as enhanced the gene expression of MITF (**C**), TRP1 (**D**), and TYR (**E**). phADM with increasing concentrations (0.3–1%) successfully reversed these changes. Data are presented as mean ± SD. * comparison to negative control, * *p* < 0.05. # comparison to α-MSH control, # *p* < 0.05. α-MSH, alpha-melanocyte stimulating hormone. MITF, microphthalmia-associated transcription factor. TRP1, tyrosinase-related protein 1. TYR, tyrosinase. phADM, particulated human acellular dermal matrix.

Stimulation with α-melanocyte-stimulating hormone (α-MSH) significantly increased both intracellular and extracellular melanin levels compared with the negative control. In contrast, phADM treatment markedly and dose-dependently suppressed melanin accumulation in both compartments (Figure 13A,B), with the greatest inhibitory effect observed at a concentration of 1%. Consistent with the reduction in melanin production, phADM significantly downregulated the expression of key melanogenesis-related genes, including *microphthalmia-associated transcription factor* (*MITF*), *tyrosinase-related protein 1* (*TRP1*), and *tyrosinase* (*TYR*), compared with the α-MSH-stimulated control. Gene expression levels decreased in a concentration-dependent manner, with the most pronounced inhibition observed at higher phADM concentrations (Figure 13C–E). Collectively, these findings suggest that phADM may attenuate melanogenesis by suppressing melanin synthesis through inhibition of the *MITF–TYR–TRP1* signaling axis.

### 2.6. Safety Profile

In the clinical trial, no serious adverse events occurred during the study, and mild events (such as transient erythema and slight swelling) resolved spontaneously without treatment.

## 3. Discussion

Skin senescence is a cumulative process driven by the interplay of intrinsic aging and extrinsic stressors. This “inflammaging” phenotype—characterized by the accumulation of senescent cells and the senescence-associated secretory phenotype (SASP)—precipitates ECM degradation, basement membrane disruption, and dysregulated melanocyte–keratinocyte signaling [22]. Clinically, these pathophysiological changes manifest as multidimensional tissue decline, including wrinkles, sagging, barrier dysfunction, pore enlargement, and pigmentary irregularities [22,23,24], prompting the research in oral, topical, and injectable rejuvenating agents [25,26,27,28]. Against this biological backdrop, therapeutic strategies that move beyond symptom-level correction toward restoration of the dermal microenvironment have become increasingly relevant, underscoring the importance of biomaterials that can reconstitute native ECM structure and function at the tissue level.

In this context, the dermis possesses distinct intrinsic characteristics that extend beyond its role as a surface covering. This includes a highly ECM microarchitecture, biomechanical support capacity, growth factor sequestration, matrikine-mediated signaling, and its function as a cellular niche supporting fibroblast adhesion, migration, angiogenesis, and tissue remodeling [29,30,31,32,33,34]. These properties fundamentally underlie the dermis’s utility in tissue reconstruction, repair, and replacement. Furthermore, particulation of dermis tissue alters only its morphological form factor while preserving its biochemical identity, ECM composition, and biofunctional attributes relevant to tissue restoration [16]. This provides the foundation to utilize phADM to comprehensively restore and repair skin function and structure.

Our findings demonstrate that phADM treatment leads to significant clinical improvements in skin density, volume, and elasticity, accompanied by marked reductions in wrinkle severity and pore size, as well as lifting effects in the periorbital and cheek regions. These clinical outcomes are mechanistically supported by the capacity of phADM to promote constructive dermal remodeling through preserved ECM architecture and active host–matrix interactions. High-magnification SEM analysis confirmed that phADM retains ultrastructural ECM features comparable to those of sheet-type hADM, a material with a long history of safe clinical application in reconstructive surgery [16,17,18,19]. Preservation of this native ECM organization enables homogeneous intradermal distribution following injection while maintaining tissue architecture, as further corroborated by histological evaluation. Importantly, this structural and biological compatibility was substantiated by in vivo evidence demonstrating active fibroblast migration and integration into the matrix, together with localized neocollagenesis at the matrix–host interface. In parallel, in vitro assays showed that phADM stimulated HDFs proliferation and enhanced the synthesis of collagen, elastin, and key angiogenic factors, including VEGF, FGF, and PDGF. These findings are consistent with prior reports indicating that phADM supports fibroblast adhesion, proliferation, and ECM deposition with favorable tolerability in soft tissue augmentation [16,17]. Collectively, these mechanisms likely account for the observed clinical benefits, supporting the interpretation that phADM undergoes minimal structural alteration during particulation and therefore retains its function as a minimally manipulated, human-derived biomaterial capable of restoring dermal biomechanics.

A critical finding of this study is the restoration of the skin barrier and basement membrane integrity. The dermal-epidermal junction (DEJ) is compromised in photoaged skin [35]. Our ex vivo UVB-irradiated model demonstrated that phADM treatment significantly upregulated key basement membrane proteins, Nidogen I and Collagen IV [36,37], indicating structural reinforcement of the DEJ. Concurrently, in vitro data showed a dose-dependent increase in HAS expression and intracellular HA content, which plays an integral part in regulating moisture [38]. These molecular changes parallel the observed reductions in TEWL and improvements in hydration in the test group, consistent with graft-based studies reporting recovery of approximately 82–86% of normal skin barrier and hydration function following acellular dermal matrix-based reconstruction [39]. As a while, these findings support a dual mechanism where phADM reinforces the physical barrier while boosting endogenous hydration capacity.

Furthermore, phADM exhibited significant depigmenting efficacy, addressing the dysregulated melanogenesis often associated with aging. While clinical results showed a progressive reduction in pigmentation area, mechanistic assays suggested that phADM downregulates the *MITF–TYR–TRP1* signaling axis, thereby suppressing melanin synthesis at the transcriptional level [40]. This anti-melanogenic effect, combined with the observed reduction in erythema, suggests that phADM may modulate the inflammatory milieu that drives post-inflammatory hyperpigmentation and uneven skin tone [41].

Uniquely, the safety profile of phADM appears distinct from synthetic biostimulators (e.g., PLLA, CaHA). While synthetic agents typically leverage a controlled inflammatory response to induce neocollagenesis [42], phADM demonstrated potent anti-inflammatory effects in activated macrophages by suppressing LPS-induced cytokines (IL-1β, TNF-α, IL-6) [43], thereby maintaining the homeostasis of the dermal microenvironment. This suggests that phADM promotes restoration through biocompatible and homeostatic tissue integration rather than inflammation-driven neocollagenesis, offering a theoretical advantage in minimizing the risk of granulomas or persistent nodules. Consistently favorable participant and investigator assessments with no observed serious adverse events further support the translational potential of phADM within the skin-booster landscape.

Despite these promising findings, several limitations warrant consideration. First, the small sample size and split-face design, while controlling for inter-individual variability, limit generalizability. Second, the 20-week follow-up precludes the assessment of long-term durability and delayed adverse events. Third, as the test product was a mixture of phADM and a non-crosslinked HA carrier, separating the synergistic effects remains challenging, although the distinct regenerative markers observed suggest phADM as the primary bioactive driver. In addition, given the relatively small sample size and the large number of evaluated clinical endpoints across multiple time points, there is a potential risk of inflated type I error due to multiple comparisons. Although post hoc corrections were applied for within-group analyses, no formal adjustment for multiplicity across all endpoints was performed. Therefore, some statistically significant findings should be interpreted with caution, and confirmation in larger, adequately powered studies is warranted. Finally, while transcriptional changes in melanogenesis were significant, protein-level validation is required to fully elucidate the pathway. Future research should involve larger, controlled trials with active comparators and advanced spatial profiling to further define the regenerative potential of injectable phADM.

## 4. Materials and Methods

### 4.1. Prospective Split-Face Clinical Trial

#### 4.1.1. Study Design and Participants

This 20-week, randomized, split-face, double-blinded, prospective clinical trial was conducted from November 2024 to May 2025 at Severence Hospital, Seoul, Republic of Korea, in accordance with the Declaration of Helsinki. The study protocol received approval from the Clinical Trial Review Board of the Severance Hospital (IRB number 1-2024-0042, date: 12 September 2024) and was registered on clinicaltrials.gov (Identifier: NCT07155278, date: 4 September 2025). A total of 20 participants were recruited in consideration of the number of subjects that can be enrolled within 3 months after IRB approval in a typical clinical setting. Inclusion criteria comprised male or female between the ages of 30 and 65, having an ACSS score of 2 to 3 (Table S1) at screening, and being available for follow-up throughout the study period. The ACSS is used to assess the skin texture, creases, and crosshatching of the cheek area [44]. Subjects were excluded if they had received or planned to receive other cosmetic procedures such as facial fillers, botulinum toxin injections, or mesotherapy within 12 months of screening. Other exclusion criteria included a history of inflammatory skin diseases, contagious diseases, skin grafts, autoimmune diseases, complex allergies, and having recently used external or oral medications for skin improvement within six months of screening. Each right or left side of the eligible participants’ faces was randomly assigned one of the two treatment arms.

#### 4.1.2. Randomization and Allocation of Participants

Randomization was conducted using the Randomization module of SAS software (version 9.4) by an independent investigator. Test products were applied to each half of the participants’ faces according to their allocated randomization numbers, ensuring that participants were blinded to treatment assignments. The analyzing investigator was also unaware of treatment groups during the assessment and comparison of skin parameters, ensuring the double-blinded design. All participants were informed about the study’s goals, procedures, and possible risks, and written informed consent was provided before their participation.

#### 4.1.3. Test Products

phADM (Elravie Re2O, L&C Bio Co., Ltd., Seoul, Republic of Korea) was manufactured using donor-derived human dermal tissue obtained with informed consent for cosmetic use. All procedures, from donor tissue procurement through processing and packaging, were conducted in strict accordance with the guidelines of the American Association of Tissue Banks (AATB). The tissue handling and manufacturing processes complied with established safety and quality standards to ensure appropriate donor screening, traceability, and sterility. As the material was derived from consented donor tissue and processed following recognized regulatory guidelines, no ethical concerns were associated with its use in this study.

The control product was a non-crosslinked HA skin booster (Elravie Balance; Humedix Co., Ltd., Gyeonggi, Republic of Korea) containing 20 mg of HA per 1.5 mL and lidocaine. The test product was prepared by mixing 150 mg of phADM with 1.5 mL of the control HA product, 2.1 mL of normal saline, and 0.4 mL of lidocaine, yielding a total volume of 4.0 mL. Consequently, the administered volume of 1.5 mL per side contained approximately 56.25 mg of phADM and 7.5 mg of HA. Elravie Re2O is a human tissue product regulated by the Ministry of Food and Drug Safety (MFDS) of Korea. It preserves the ECM structure, consisting of approximately 80% collagen, 3% elastin, and 0.4% sGAGs, with a residual DNA content of <50 ng/mg. In a split-face design, a blinded board-certified dermatologist administered 1.5 mL of the test product to one side of the face and 1.5 mL of the control product to the other. To ensure consistent injection depth and dosing accuracy, an automated injection device (Dermashine; Huons Meditech Co., Ltd., Seongnam, Republic of Korea) was employed, targeting the mid-dermis layer.

To evaluate the preservation of ECM architecture in phADM, SEM was employed. phADM and a commercially available sheet-type human acellular dermal matrix (hADM; MegaDerm^®^, L&C Bio Co., Ltd., Seoul, Republic of Korea) were sputter-coated with a thin carbon layer using an ion sputter coater (EM ACE600; Leica Microsystems, Wetzlar, Germany). Surface ultrastructure was subsequently examined using a field-emission scanning electron microscope (MERLIN; Zeiss, Oberkochen, Germany).

#### 4.1.4. Skin Measurements

Skin measurements were conducted at baseline (Visit 1) and at 1, 4, 8, 12, 16, and 20 weeks post-treatment (Visits 2–7). For each measurement session, participants’ faces were first cleansed, then they were acclimatized for 30 min in a controlled environment maintained at a temperature of 20–24 °C and 45–55% relative humidity. Subsequently, we used various devices to quantitatively measure specific skin indices:

- Nasolabial fold depth, pigmentation area, and pore area were measured using the Antera 3D CS (Miravex, Dublin, Ireland), which captures 3D surface images and quantifies wrinkle depth in millimeters (mm), pigmentation and pore area in mm^2^.
- Infraorbital wrinkles were measured using the Eve V (EVELAB INSIGHT, Singapore) in pixels. The device utilizes a detailed 3D assessment of facial conditions through contour evaluation and high-resolution imaging.
- Skin volume was measured in cubic millimeters (mm^3^) with the 3D LifeViz micro (QuantifiCare, Biot, France), a non-contact device utilizing structured light projection.
- Skin density was evaluated in percentage (%) using the Skin Scanner (tpm, Luneburg, Germany), which employs a 22 MHz ultrasound transducer to generate density images.
- Eye area lifting and cheek area lifting were assessed with the Morpheus3D (Morpheus, Gyeonggi, Republic of Korea), a medical image analysis device that measures the increase in curve length in millimeters (mm) to determine the degree of lifting. Specifically, the eye lifting effect is measured as the curve distance from the eye corner to the ear tragus, while the cheek lifting influence is calculated as the curve length from the side of the nose to the ear tragus.
- Erythema (a-value) was measured using the VISIA CR (Canfield, NJ, USA), a device capturing standardised, high-resolution digital images of the entire face using different light sources.
- Skin hydration (gray index) was assessed using Moisture Map MM 100 Courage & Khazaka, Köln, Germany), which uses capacitance imaging to detect the distribution of moisture.
- TEWL (g/m^2^/h) was assessed using the Tewameter (Courage & Khazaka, Köln, Germany), which measures the density gradient of the water evaporation from the skin by a temperature sensor and a humidity sensor.
- Skin elasticity was evaluated using the Cutometer (Courage & Khazaka, Köln, Germany). The device measures skin elasticity via a suction phase lasting 2 s, followed by a 2-s relaxation phase. R2 (Visco-elasticity) is defined as the resistance to the mechanical force versus the ability to return.

Furthermore, the overall aesthetic improvement was subjectively evaluated by both participants and evaluators using the GAIS, which scores improvement relative to the pre-treatment state (Table S2). Alterations in the evaluator-assessed ACSS score were also recorded.

#### 4.1.5. Adverse Event Assessment

The safety of the test products was assessed throughout the study period. During each visit, participants were monitored for any adverse reactions, and the use of any concomitant medications that could potentially affect the study was checked. All confirmed and reported adverse reactions from all subjects were compiled to determine the incidence rate, which was then used as data for the overall safety evaluation of the product.

### 4.2. Ex Vivo Human Skin Model

#### 4.2.1. Human Skin Tissue Culture and Histological Analysis

Human skin tissue experiments were approved by the Global Medical Research Center Institutional Review Board (IRB No. GIRB-25613-OH, date: 25 June 2025). Skin tissues were collected from a 49-year-old Asian female donor, and each ex vivo experiment was repeated three times. Subsequently, two experimental groups were evaluated: a control and a phADM-treated group. The latter received a single intradermal injection of 0.1 mL at a uniform depth using a 27-gauge needle.

24 h after treatment, the human skin tissues were harvested, fixed in 10% neutral-buffered formalin, and paraffin-embedded for subsequent histological comparison between groups. Paraffin-embedded sections were mounted on glass slides, deparaffinized, and subjected to hematoxylin and eosin (H&E) staining. Briefly, nuclei were stained with hematoxylin (S3309; Dako, Glostrup, Denmark), rinsed thoroughly with running water, and counterstained with eosin (318906; Sigma-Aldrich, St. Louis, MO, USA) to visualize cytoplasmic structures. Following staining, the sections were washed, fully dehydrated, and mounted using a permanent mounting medium. Histological images of the epidermis and dermis were acquired using a light microscope (BX43F; Olympus, Tokyo, Japan) at magnifications of 40× or 400×. In phADM-injected tissues, histological differences were evaluated by comparing phADM-treated regions with adjacent untreated peripheral regions.

#### 4.2.2. UVB Irradiation Protocol and Immunofluorescence Evaluation of Basement Membrane-Associated Proteins

To assess the preventive effects of phADM against UVB-induced basement membrane disruption, an independent ex vivo experiment incorporating controlled UVB irradiation was performed. The human skin tissues from the previously mentioned donor were randomly assigned to three groups: a negative control, a UVB-irradiated control, and a UVB + phADM–treated group. phADM was prepared at a concentration of 30 mg/mL and administered as a single intradermal injection (0.1 mL) at a consistent depth using a 27-gauge needle. Following treatment, tissues were exposed to UVB irradiation using a UV cross-linker (BLX 312; Vilber Lourmat) at a wavelength of 312 nm, delivering a total dose of 300 mJ/cm^2^ once daily for three consecutive days.

72 h after the final irradiation, the human skin samples were harvested, embedded in optimal cutting temperature compound, and cryosectioned at a thickness of 6 µm using a cryostat microtome (Leica Biosystems, Nußloch, Germany). Sections were mounted on silane-coated glass slides and incubated with primary antibodies against Nidogen-1 (MAB2570-100; R&D Systems, Minneapolis, MN, USA) and Collagen IV (ab6586; Abcam, Cambridge, UK). After washing, sections were incubated with fluorescence-conjugated secondary antibodies (Goat pAb to Rabbit IgG [H + L], A11012; Goat pAb to Mouse IgG [H + L], A11001; Invitrogen, Carlsbad, CA, USA), followed by nuclear counterstaining using a DAPI-containing mounting medium (VECTASHIELD^®^ HardSet Antifade Mounting Medium with DAPI; H-1500-10; Vector Laboratories, Newark, CA, USA). Fluorescent images were acquired at 200× magnification using a confocal laser scanning microscope (LSM700; Zeiss, Oberkochen, Germany). Quantitative analysis was performed using Zen image analysis software (version 3.13, Zeiss) by measuring fluorescence intensity normalized to basement membrane length, with higher values indicating increased expression of basement membrane-associated proteins. Each experiment is conducted three times.

### 4.3. In Vivo Rat Model

#### 4.3.1. Animal Study Design

The animal studies were performed after receiving approval of the Institutional Animal Care and Use Committee (IACUC) of Hulux Inc. (Approval No. Hulux-2025-02-006, approval date: 23 January 2025).

Male Sprague–Dawley (SD) rats (7 weeks old) were obtained from RaonBio Inc. (Republic of Korea). Upon arrival, animals were inspected for general health status, body weight, and overall condition and then acclimated for 7 days before experimentation. Rats were housed in polystyrene cages (W 277 × L 423 × H 194 mm) with two animals per cage under controlled environmental conditions: room temperature of 23 ± 3 °C, relative humidity of 55 ± 15%, and 10–20 air changes per hour. A 12 h light/dark cycle was maintained (lights on at 07:00 AM and off at 07:00 PM) with an illuminance of 150–300 lux. Standard chow (Teklad Certified Irradiated Global 18% Protein Rodent Diet 2018C, Envigo RMS Inc., Indianapolis, IN, USA) and UV-sterilized tap water were provided ad libitum. Cages were replaced once weekly, and water bottles were replaced at least twice per week. Animals were identified by tail marking using black, red, or blue permanent markers, and identification cards were attached to each cage.

After acclimation, a total of 4 rats were used for subsequent experiments. 5% phADM was administered via a single intradermal injection in separate disposable syringes into the dorsal region (center of the back) under inhalation anesthesia with isoflurane. Each animal received 2 injections on their dorsum, allowing for intra-individual comparison. The dosing volume for each implantation site was set at 50 µL, as determined in consultation with the study sponsor. General clinical observations were conducted once daily to monitor health status. 1 week after implantation, animals were anesthetized with isoflurane. The implanted areas, including the surrounding skin tissue, were excised. Animals were then euthanized by exsanguination following abdominal opening and severing of the cardiac vessels under deep anesthesia. Collected tissue samples were fixed in 4% paraformaldehyde and subsequently processed for histopathological evaluation.

#### 4.3.2. Histopathological Analysis

For histological evaluation, tissues were fixed in 4% paraformaldehyde and processed into paraffin blocks. Paraffin sections were mounted onto glass slides, deparaffinized, and stained according to the procedures described below. After staining, the sections were washed, fully dehydrated, and mounted using a mounting solution. The prepared slides were examined and imaged using a light microscope (MD3000LED; Leica Microsystems, Wetzlar, Germany) at magnifications of 5× or 400×.

#### 4.3.3. H&E Staining

H&E staining was performed to evaluate cellular infiltration in rat skin tissues injected with Re2O. Deparaffinized tissue sections were stained with hematoxylin (104302; Merck, Rahway, NJ, USA) to visualize cell nuclei, followed by rinsing under running water. The cytoplasm was subsequently stained with eosin (230251; Sigma-Aldrich), after which the sections were washed and fully dehydrated. Cell numbers were quantified from the acquired histological images and normalized to unit area to assess cellular infiltration. A higher number of cells per unit area was interpreted as increased cellular density within the tissue sections.

#### 4.3.4. HV Staining

Herovici staining was conducted to evaluate newly synthesized collagen in rat skin tissues injected with phADM. The staining was performed using a Herovici’s Stain Kit (ScyTek Laboratories, Inc., Logan, UT, USA) according to the manufacturer’s instructions. Histological images were analyzed using image analysis software (Zen image analysis; Zeiss). The total tissue area and the area of newly formed collagen (blue-stained regions) were quantified based on pixel values. The extent of neocollagen formation was expressed as the ratio of blue-stained collagen area to the total tissue area. A higher proportion of blue-stained regions indicated greater neocollagen deposition within the tissue sections.

### 4.4. In Vitro Models

#### 4.4.1. Cell Culture

HDFs (Thermo Fisher Scientific, Waltham, MA, USA), used for the evaluation of anti-wrinkle efficacy; mouse melanoma cells (B16F10, American Type Culture Collection, Manassas, VA, USA), used for the evaluation of skin-whitening efficacy; and mouse macrophages (RAW 264.7, American Type Culture Collection), used for the evaluation of anti-inflammatory efficacy, were cultured in Dulbecco’s Modified Eagle Medium (DMEM; Lonza, Walkersville, MD, USA) supplemented with 10% fetal bovine serum (FBS; Gibco, Waltham, MA, USA) and 1% penicillin–streptomycin (Gibco). HEKs (Thermo Fisher Scientific), used for the evaluation of moisturizing efficacy, were cultured in EpiLife™ Medium (Gibco) supplemented with Human Keratinocyte Growth Supplement (Gibco). All cells were maintained at 37 °C in a humidified atmosphere containing 5% CO_2_. HDFs were used in passage 6; mouse melanoma cells were used in passage 9; mouse macrophages were used in passage 8; HEKs were used in passage 4.

#### 4.4.2. Cell Viability and Proliferation Assay

Cultured cells were seeded into 96-well plates at a density of 5 × 10^3^ cells/well in 100 µL of culture medium. After 24 h, when cell confluence exceeded 80%, the test product (0.001%, 0.01%, 0.1%, 0.3%, 0.5%, 1%) was applied under serum-free conditions. Following incubation for 24, 48, or 72 h, the culture medium was removed, and the cells were washed with Dulbecco’s phosphate-buffered saline (DPBS). A WST substrate solution (Cell Counting Kit-8 [CCK-8]; Dojindo, Kumamoto, Japan) was prepared by mixing with culture medium at a 1:9 ratio, and 100 µL was added to each well. The plates were incubated at 37 °C for 2 h, after which absorbance was measured at 450 nm using a microplate reader (Varioskan™ LUX; Thermo Fisher Scientific).

Measured OD values were corrected by subtracting blank values, and cell viability was expressed as a percentage relative to the negative control, which was set at 100%. Higher absorbance values indicated greater numbers of viable cells.

#### 4.4.3. Protein Extraction

Cells were seeded into 6-well plates at densities of 1 × 10^5^ cells/well for HDFs and 5 × 10^4^ cells/well for mouse macrophages and HEKn, with 2 mL of culture medium per well. After 24 h, when the 3 types of cell confluence exceeded 80%, the test products (0.3%, 0.5%, 1%) were applied in serum-free medium. For mouse macrophages, lipopolysaccharide (LPS; 1 µg/mL) was co-treated to induce an inflammatory response. L-NMMA (100 µM; Sigma-Aldrich, M7033) was used as the positive control.

After 24 h of treatment, extracellular proteins (collagen type I, collagen type III, elastin, VEGF, FGF, PDGF, TGF-β1, IL-1β, IL-6, TNF-α, and PGE-2) were collected from the culture supernatants and centrifuged at 2000× *g* for 10 min to remove cellular debris. The clarified supernatants were used for subsequent analyses.

For intracellular protein (HA and HAS) extraction, culture media were removed and cells were lysed with 250 µL of Pro-PREP™ Protein Extraction Solution (iNtRON Biotechnology, Gyeonggi-do, Republic of Korea). Cells were detached using a scraper, followed by centrifugation at 2000× *g* for 10 min, and the resulting supernatants were collected. Protein concentrations were determined using a Bicinchoninic Acid (BCA) Protein Assay Kit (Sigma-Aldrich).

#### 4.4.4. Enzyme-Linked Immunosorbent Assay (ELISA)

ELISA was performed to quantify the production of proteins associated with anti-wrinkle efficacy—collagen type I, collagen type III, elastin, VEGF, FGF, PDGF, and TGF-β1; moisturizing efficacy—HA and HAS2; and anti-inflammatory efficacy—IL-1β, IL-6, TNF-α, and PGE-2.

Protein levels were measured in the extracted samples using commercially available ELISA kits (Table S3), following the manufacturer’s instructions. Absorbance was measured using a microplate reader (Varioskan™ LUX; Thermo Fisher Scientific, Waltham, MA, USA). Protein concentrations were calculated by substituting the measured optical density (OD) values into regression equations derived from standard curves. Higher absorbance values indicated greater protein abundance.

#### 4.4.5. Melanin Content Assay

This assay was conducted to quantify intracellular and extracellular melanin production following treatment with the test product. Mouse melanoma cells were seeded in 6-well plates at 1 × 10^5^ cells/well and cultured for 24 h until >80% confluence. Cells were then stimulated with α-melanocyte-stimulating hormone (α-MSH, 100 nM; Sigma-Aldrich, M4135) and co-treated with the test product (0.1%, 0.3%, or 0.5%) in phenol red–free DMEM containing 10% FBS for 72 h.

For intracellular melanin analysis, the culture medium was removed and cells were lysed with 250 µL of 1 M NaOH containing 10% DMSO. After incubation at 60 °C for 10 min under light-protected conditions, cells were scraped and further incubated at 95 °C for 30 min. Lysates were centrifuged at 4000× *g* for 10 min, and the supernatants were collected. For extracellular melanin analysis, culture supernatants were centrifuged at 4000× *g* for 10 min, and the clarified supernatants were collected.

Absorbance of intracellular and extracellular samples was measured at 475 nm using a microplate reader (Varioskan™ LUX; Thermo Fisher Scientific). Protein concentrations were determined using a bicinchoninic acid (BCA) protein assay kit (Sigma-Aldrich), with higher absorbance values indicating increased melanin production.

#### 4.4.6. Real-Time Polymerase Chain Reaction (RT-PCR)

Real-time PCR was performed to assess the mRNA expression of pigmentation-related genes, including *MITF*, *TRP1*, and *TYR*. Mouse melanoma cells were seeded in 6-well plates at 1 × 10^5^ cells/well and cultured for 24 h until >80% confluence. Cells were then stimulated with α-MSH (100 nM) and treated with the test product in DMEM supplemented with 10% fetal bovine serum for an additional 24 h.

Total RNA was extracted using TRIzol™ Reagent (Invitrogen) according to the manufacturer’s instructions. Equal amounts of RNA were reverse-transcribed into cDNA using the RNA to cDNA EcoDry™ Premix (Oligo dT) (Clontech, Mountain View, CA, USA). Quantitative PCR was performed using TaqMan™ Fast Advanced Master Mix (Applied Biosystems, Waltham, MA, USA) and gene-specific TaqMan™ probes for MITF (Mm00434954_m1), TRP1 (Mm00453201_m1), and TYR (Mm00495817_m1). The amplification protocol consisted of an initial hold step followed by 40 cycles of denaturation and annealing/extension. Gene expression levels were normalized to GAPDH (Mm99999915_g1; Applied Biosystems) and calculated using the comparative C_t_ method. Lower C_t_ values indicated higher transcript abundance.

### 4.5. Statistical Analysis

Statistical analysis was performed using the IBM SPSS Statistics 27.0 program, with a significance level set at *p* < 0.05. Data were reported as mean ± standard deviation (SD). For within-group comparisons, one-way repeated measures ANOVA was used for data that satisfied the normality test, with within-group contrasts to identify differences between time points. For non-normally distributed data, the non-parametric Friedman test was performed, followed by a Wilcoxon signed-rank test with Bonferroni correction for post hoc analysis. For comparisons of the change from baseline between groups, we used the paired *t*-test for normally distributed data and the Wilcoxon signed rank test for non-normally distributed data. For comparisons of the ACSS and GAIS between groups, we used the Wilcoxon signed rank test.

## 5. Conclusions

In conclusion, injectable phADM showed promising clinical and biological signals suggestive of regenerative dermal remodeling in this integrated preclinical and randomized split-face clinical study. While these findings indicate potential benefits across structural, functional, and pigmentation-related parameters, the limited sample size warrants cautious interpretation. Larger, adequately powered studies are needed to validate its efficacy and durability.

## Figures and Tables

**Table 1:** Baseline characteristics of participants.

Characteristics	Values
Age (Mean ± SD)	54.7 ± 9.246
Gender	
Female	13
Male	7
ACSS score (Mean ± SD)	
Test group	2.9 ± 0.3
Control group	2.9 ± 0.3
*p*-value ^1^	0.999

^1^ Wilcoxon signed-rank test.

**Table 2:** Evaluation of skin parameters.

Group	Baseline	4 Weeks	8 Weeks	12 Weeks	16 Weeks	20 Weeks
Skin density (%)						
Test	65.185 ± 6.316	72.763 ± 6.265 ^b,d^	72.333 ± 5.042 ^b^	75.063 ± 5.272 ^b,d^	75.355 ± 4.926 ^b,d^	75.154 ± 5.189 ^b,d^
Control	65.998 ± 5.680	70.281 ± 6.544 ^b^	70.809 ± 5.342 ^b^	71.601 ± 5.254 ^b^	71.255 ± 5.781 ^b^	71.789 ± 6.423 ^b^
Skin volume (mm^3^)						
Test	298.028 ± 83.400	319.202 ± 83.400 ^b,d^	325.484 ± 84.122 ^b,d^	343.356 ± 82.662 ^b,d^	348.330 ± 84.157 ^b,d^	332.205 ± 83.778 ^b,d^
Control	295.788 ± 85.941	300.842 ± 88.499	304.840 ± 90.700	306.476 ± 91.925	305.642 ± 91.962	297.300 ± 91.016
TEWL (g/m^2^/h)						
Test	14.682 ± 3.294	13.895 ± 2.873	13.010 ± 2.621 ^b,c^	13.190 ± 2.947 ^b^	13.877 ± 3.358 ^a^	13.092 ± 2.625 ^b,c^
Control	14.950 ± 2.615	14.622 ± 2.910	13.985 ± 2.358	14.138 ± 2.594	14.392 ± 2.533	14.482 ± 2.831
Skin hydration (grey index)						
Test	114.345 ± 21.678	132.238 ± 18.236 ^b^	136.286 ± 16.248 ^b,c^	140.037 ± 17.909 ^b,c^	139.614 ± 15.573 ^b,d^	138.869 ± 14.275 ^b,d^
Control	117.206 ± 21.243	128.500 ± 17.340 ^a^	131.088 ± 16.710 ^b^	134.869 ± 16.718 ^b^	132.682 ± 16.528 ^b^	133.737 ± 13.324 ^b^
Pore area (mm^2^)						
Test	61.985 ± 28.812	51.034 ± 23.784 ^b^	45.888 ± 29.849 ^b^	44.944 ± 28.865 ^b,c^	42.756 ± 26.464 ^b,d^	41.908 ± 28.787 ^b,c^
Control	58.311 ± 28.865	50.175 ± 25.713 ^b^	48.660 ± 26.619 ^b^	48.468 ± 25.404 ^b^	49.763 ± 26.379 ^b^	48.574 ± 25.598 ^b^
Skin elasticity (R2)						
Test	0.586 ± 0.074	0.643 ± 0.075 ^b,c^	0.633 ± 0.065 ^b^	0.639 ± 0.070 ^b^	0.641 ± 0.059 ^b^	0.651 ± 0.071 ^b,c^
Control	0.598 ± 0.060	0.635 ± 0.061 ^b^	0.633 ± 0.052 ^b^	0.634 ± 0.047 ^b^	0.643 ± 0.046 ^b^	0.642 ± 0.048 ^b^
Nasolabial fold depth (mm)						
Test	0.169 ± 0.071	0.160 ± 0.065 ^b,d^	0.156 ± 0.063 ^b,d^	0.153 ± 0.063 ^b,d^	0.149 ± 0.061 ^b,c^	0.146 ± 0.060 ^b,c^
Control	0.159 ± 0.044	0.154 ± 0.042 ^b^	0.152 ± 0.042	0.149 ± 0.042 ^b^	0.146 ± 0.042 ^b^	0.143 ± 0.040 ^b^
Infraorbital wrinkles (pixels)						
Test	2838.900 ± 1362.696	2461.500 ± 1177.704 ^b^	2325.900 ± 1082.646 ^b,c^	2254.050 ± 1045.049 ^b,d^	2154.000 ± 889.961 ^b,d^	2238.050 ± 897.905 ^b,d^
Control	2805.050 ± 1251.803	2482.200 ± 1165.511 ^b^	2480.050 ± 1182.191 ^b^	2440.300 ± 979.397 ^b^	2438.050 ± 1044.166 ^b^	2504.600 ± 1102.412 ^b^
Eye lifting (mm)						
Test	120.705 ± 5.909	121.635 ± 6.194 ^b,c^	121.705 ± 5.911 ^b,d^	122.120 ± 5.427 ^b,c^	122.640 ± 5.677 ^b,d^	122.890 ± 5.611 ^b,d^
Control	120.230 ± 6.270	120.805 ± 6.598	120.505 ± 6.116	120.710 ± 6.368	120.825 ± 6.437	120.890 ± 6.674
Cheek lifting (mm)						
Test	44.651 ± 7.878	45.275 ± 8.004 ^b^	45.276 ± 8.164 ^b^	45.117 ± 8.075 ^b,c^	45.106 ± 8.085 ^b,d^	45.476 ± 7.986 ^b,d^
Control	41.269 ± 8.479	41.700 ± 8.474 ^b^	41.626 ± 8.493 ^b^	41.423 ± 8.563	41.404 ± 8.479 ^a^	41.789 ± 8.525 ^b^
Pigmentation area (mm^2^)						
Test	26.850 ± 12.671	24.050 ± 10.118 ^b^	22.400 ± 9.087 ^b,c^	22.650 ± 10.080 ^b,d^	23.100 ± 11.097 ^b,c^	22.950 ± 11.307 ^b,c^
Control	22.600 ± 8.262	21.300 ± 8.014 ^b^	20.750 ± 8.252 ^b^	20.500 ± 8.345 ^b^	20.050 ± 7.824 ^b^	19.900 ± 8.039 ^b^
Erythema (a-value)						
Test	10.151 ± 2.895	10.168 ± 2.685	9.836 ± 2.794	9.671 ± 2.834 ^c^	9.726 ± 2.904 ^c^	9.496 ± 2.610 ^c^
Control	10.061 ± 2.715	10.048 ± 2.746	9.951 ± 2.791	10.208 ± 3.356	10.320 ± 3.164	10.425 ± 3.504
ACSS score						
Test	2.900 ± 0.308	2.000 ± 0.459 ^b,d^	2.000 ± 0.459 ^b,d^	1.950 ± 0.394 ^b,d^	1.850 ± 0.489 ^b,d^	1.900 ± 0.553 ^b,d^
Control	2.900 ± 0.308	2.900 ± 0.308	2.850 ± 0.489	2.850 ± 0.489	2.800 ± 0.523	2.800 ± 0.523
Investigator GAIS						
Test	-	3.500 ± 0.607	4.050 ± 0.510 ^f^	4.250 ± 0.550 ^e^	4.500 ± 0.513 ^f^	4.450 ± 0.510 ^f^
Control	-	3.150 ± 0.671	3.550 ± 0.759	3.850 ± 0.745	4.000 ± 0.795	3.850 ± 0.671
Participant GAIS						
Test	-	3.900 ± 0.447	4.250 ± 0.716 ^e^	4.350 ± 0.587 ^e^	4.350 ± 0.587 ^f^	4.500 ± 0.513 ^f^
Control	-	3.700 ± 0.571	3.550 ± 0.759	3.950 ± 0.510	3.850 ± 0.489	3.850 ± 0.366

Data are presented as mean ± SD. ^a,b^ comparison within groups by Repeated Measures ANOVA or Friedman test (followed by post hoc Wilcoxon signed rank test with Bonferroni correction), depending on normality. ^a^ *p* < 0.05; ^b^ *p* < 0.01. ^c,d^ comparison of the change from baseline between groups by paired *t*-test or Wilcoxon signed rank test, depending on normality. ^c^ *p* < 0.05; ^d^ *p* < 0.01. ^e,f^ comparison between groups by the Wilcoxon signed rank test. ^e^ *p* < 0.05; ^f^ *p* < 0.01. TEWL, transepidermal water loss; ACSS, Allergan Cheek Smoothness Scale; GAIS, Global Aesthetic Improvement Scale.

## Data Availability

The authors confirm that the data supporting the findings of this study are available within the article and its Supplementary Materials.

## Supplementary Materials

The following supporting information can be downloaded at: https://www.mdpi.com/article/10.3390/ijms27052193/s1.

## Abbreviations

The following abbreviations are used in this manuscript:

ACSS	Allergan Cheek Smoothness Scale
ADM	Acellular Dermal Matrix
AATB	American Association of Tissue Banks
α-MSH	Alpha-melanocyte-stimulating hormone
BCA	Bicinchoninic Acid
CaHA	Calcium Hydroxylapatite
CCK-8	Cell Counting Kit-8
CO_2_	Carbon dioxide
DAPI	4′,6-diamidino-2-phenylindole
DEJ	Dermal–epidermal junction
DMEM	Dulbecco’s Modified Eagle Medium
DMSO	Dimethyl sulfoxide
DPBS	Dulbecco’s Phosphate-Buffered Saline
ECM	Extracellular Matrix
ELISA	Enzyme-Linked Immunosorbent Assay
FBS	Fetal Bovine Serum
FGF	Fibroblast Growth Factor
GAIS	Global Aesthetic Improvement Scale
GAPDH	Glyceraldehyde-3-phosphate dehydrogenase
HA	Hyaluronic Acid
HAS	Hyaluronan Synthase
HAS2	Hyaluronan Synthase 2
HDF	Human Dermal Fibroblast
HEK	Human Epidermal Keratinocyte
H&E	Hematoxylin and Eosin
hADM	Human Acellular Dermal Matrix
HV	Herovici
IACUC	Institutional Animal Care and Use Committee
IL-1β	Interleukin-1 beta
IL-6	Interleukin-6
IRB	Institutional Review Board
L-NMMA	NG-methyl-L-arginine acetate salt
LPS	Lipopolysaccharide
MFDS	Ministry of Food and Drug Safety
MITF	Microphthalmia-associated Transcription Factor
OD	Optical Density
PCR	Polymerase Chain Reaction
PDGF	Platelet-Derived Growth Factor
PGE-2	Prostaglandin E_2_
phADM	Particulated Human Acellular Dermal Matrix
PLLA	Poly-L-lactic Acid
RT-PCR	Real-Time Polymerase Chain Reaction
SASP	Senescence-Associated Secretory Phenotype
SD	Sprague–Dawley
SEM	Scanning Electron Microscopy
TEWL	Transepidermal Water Loss
TGF-β1	Transforming Growth Factor-beta 1
TNF-α	Tumor Necrosis Factor-alpha
TRP1	Tyrosinase-Related Protein 1
TYR	Tyrosinase
UVB	Ultraviolet B
VEGF	Vascular Endothelial Growth Factor
WST	Water-Soluble Tetrazolium

## References

[1] Arora G., Arora S., Sadoughifar R., Batra N. (2021). Biorevitalization of the skin with skin boosters: Concepts, variables, and limitations. J. Cosmet. Dermatol..

[2] Rho N.K., Kim H.S., Kim S.Y., Lee W. (2024). Injectable “Skin Boosters” in Aging Skin Rejuvenation: A Current Overview. Arch. Plast. Surg..

[3] de Castro Costa M., Andrade C.A., Dantas R.V.F., Germani M., Buzalaf M.A.R., Soares D.G. (2025). Clinical Durability of Hyaluronic Acid-Based Dermal Fillers for Facial Application: A Systematic Review. Aesthetic Plast. Surg..

[4] Kunzler C., Hartmann C., Nowag B., Shah R., El-Banna R., Backfisch S., Schafer D., Hengl T., Hagedorn N. (2023). Comparison of Physicochemical Characteristics and Biostimulatory Functions in Two Calcium Hydroxyapatite-Based Dermal Fillers. J. Drugs Dermatol..

[5] La Gatta A., Salzillo R., Catalano C., D’Agostino A., Pirozzi A.V.A., De Rosa M., Schiraldi C. (2019). Hyaluronan-based hydrogels as dermal fillers: The biophysical properties that translate into a “volumetric” effect. PLoS ONE.

[6] Mecani R., Amiri M., Kadouch J., Sajic D., Lin F., Cheung J., Barrera D., Haroon O., Sil-Zavaleta S., Chao Y. (2025). Combined and Hybrid Treatments of Hyaluronic Acid (HA) and Calcium Hydroxylapatite (CaHA): A Systematic Review of Mechanisms of Action, Aesthetic Effectiveness, Satisfaction, and Safety Profile. Aesthetic Plast. Surg..

[7] Stein P., Vitavska O., Kind P., Hoppe W., Wieczorek H., Schurer N.Y. (2015). The biological basis for poly-L-lactic acid-induced augmentation. J. Dermatol. Sci..

[8] Alijotas-Reig J., Fernandez-Figueras M.T., Puig L. (2013). Inflammatory, immune-mediated adverse reactions related to soft tissue dermal fillers. Semin. Arthritis Rheum..

[9] Nonhoff M., Puetzler J., Hasselmann J., Fobker M., Gosheger G., Schulze M. (2024). The Potential for Foreign Body Reaction of Implanted Poly-L-Lactic Acid: A Systematic Review. Polymers.

[10] Pavicic T. (2013). Calcium hydroxylapatite filler: An overview of safety and tolerability. J. Drugs Dermatol..

[11] Vleggaar D., Fitzgerald R., Lorenc Z.P. (2014). Understanding, avoiding, and treating potential adverse events following the use of injectable poly-L-lactic acid for facial and nonfacial volumization. J. Drugs Dermatol..

[12] Gierek M., Labus W., Kitala D., Lorek A., Ochala-Gierek G., Zagorska K.M., Waniczek D., Szyluk K., Niemiec P. (2022). Human Acellular Dermal Matrix in Reconstructive Surgery—A Review. Biomedicines.

[13] Lee J.H., Kim H.G., Lee W.J. (2015). Characterization and tissue incorporation of cross-linked human acellular dermal matrix. Biomaterials.

[14] Girardeau-Hubert S., Lynch B., Zuttion F., Label R., Rayee C., Brizion S., Ricois S., Martinez A., Park E., Kim C. (2022). Impact of microstructure on cell behavior and tissue mechanics in collagen and dermal decellularized extra-cellular matrices. Acta Biomater..

[15] Stefanelli V., Lombardi J., Ferrer J., Gardocki-Sandor M. (2025). Vascularization of Human Acellular Dermal Matrices: A Comparative Study in a Nonhuman Primate Model. Tissue Eng. Part. A.

[16] Park T.H., Choi W.Y., Lee J.H., Lee W.J. (2017). Micronized Cross-Linked Human Acellular Dermal Matrices: An Effective Scaffold for Collagen Synthesis and Promising Material for Tissue Augmentation. Tissue Eng. Regen. Med..

[17] Ko H., Kim D., Shin C., Gong N.Y., You B., Oh H.S., Lee J., Oh S.H. (2023). In Vivo Efficacy of an Injectable Human Acellular Dermal Matrix. Aesthetic Plast. Surg..

[18] Maloney B.P., Murphy B.A., Cole H.P. (2004). Cymetra. Facial Plast. Surg..

[19] Sclafani A.P., Romo T., Jacono A.A., McCormick S., Cocker R., Parker A. (2000). Evaluation of acellular dermal graft in sheet (AlloDerm) and injectable (micronized AlloDerm) forms for soft tissue augmentation. Clinical observations and histological analysis. Arch. Facial Plast. Surg..

[20] Qu S., Yi J., Chen Z., Zhou J. (2021). A Potential Filling Material for Wound Healing and Shaping: Acellular Dermal Matrix Combined with Autologous Dermis. Aesthetic Plast. Surg..

[21] Zhang X., Deng Z., Wang H., Yang Z., Guo W., Li Y., Ma D., Yu C., Zhang Y., Jin Y. (2009). Expansion and delivery of human fibroblasts on micronized acellular dermal matrix for skin regeneration. Biomaterials.

[22] Lee Y.I., Choi S., Roh W.S., Lee J.H., Kim T.G. (2021). Cellular Senescence and Inflammaging in the Skin Microenvironment. Int. J. Mol. Sci..

[23] Lee S.J., Seok J., Jeong S.Y., Park K.Y., Li K., Seo S.J. (2016). Facial Pores: Definition, Causes, and Treatment Options. Dermatol. Surg..

[24] Shin S.H., Lee Y.H., Rho N.K., Park K.Y. (2023). Skin aging from mechanisms to interventions: Focusing on dermal aging. Front. Physiol..

[25] Monteil C., Barreto-Campos V., Lain E., Guillou E., Doat G., Nocera T. (2025). Enhancing Facial Rejuvenation Outcomes With a Novel Retinaldehyde-Based Cream: A Comparative Randomized Intra-Individual Study. J. Cosmet. Dermatol..

[26] Nguyen N.H., Lee Y.I., Chau N.H., Lee S.J., Kim I., Kim J., Baek K.S., Lee J.H. (2025). Porcine placenta peptides as a complementary functional food for skin rejuvenation: A 12-week randomized, double-blind, placebo-controlled trial. Complement. Ther. Med..

[27] Nguyen N.H., Lee Y.I., Jang Y.S., Lee H.K., Jung I., Lee S.J., Lee J.H. (2025). In vitro and in vivo exploration of microbiome-derived yeast extract for anti-aging and skin rejuvenation. BMC Microbiol..

[28] Chang N.W.M., Chan E.W.S. (2026). Preliminary Investigation and Safety Profile of a Novel Hybrid Filler (Hyaluronic Acid-Calcium Hydroxylapatite) in Asian Facial Rejuvenation. Plast. Reconstr. Surg. Glob. Open.

[29] Gattazzo F., Urciuolo A., Bonaldo P. (2014). Extracellular matrix: A dynamic microenvironment for stem cell niche. Biochim. Biophys. Acta.

[30] Sivaraman K., Shanthi C. (2018). Matrikines for therapeutic and biomedical applications. Life Sci..

[31] Pfisterer K., Shaw L.E., Symmank D., Weninger W. (2021). The Extracellular Matrix in Skin Inflammation and Infection. Front. Cell Dev. Biol..

[32] Boraldi F., Lofaro F.D., Bonacorsi S., Mazzilli A., Garcia-Fernandez M., Quaglino D. (2024). The Role of Fibroblasts in Skin Homeostasis and Repair. Biomedicines.

[33] Libby J.R., Royce H., Walker S.R., Li L. (2024). The role of extracellular matrix in angiogenesis: Beyond adhesion and structure. Biomater. Biosyst..

[34] Zhao T., Huang Y., Zhu J., Qin Y., Wu H., Yu J., Zhai Q., Li S., Qin X., Wang D. (2025). Extracellular Matrix Signaling Cues: Biological Functions, Diseases, and Therapeutic Targets. MedComm.

[35] Baumann L. (2007). Skin ageing and its treatment. J. Pathol..

[36] Hirakawa Y., Futaki S., Furukawa F., Kondo Y., Moriwaki S. (2020). Acute changes in nidogen-1 expression in the epidermal basement membrane of a 3-dimensional cultured human skin model after ultraviolet B irradiation. Photodermatol. Photoimmunol. Photomed..

[37] Abreu-Velez A.M., Howard M.S. (2012). Collagen IV in Normal Skin and in Pathological Processes. N. Am. J. Med. Sci..

[38] Chylinska N., Maciejczyk M. (2025). Hyaluronic Acid and Skin: Its Role in Aging and Wound-Healing Processes. Gels.

[39] Park J.Y., Lee T.G., Kim J.Y., Lee M.C., Chung Y.K., Lee W.J. (2014). Acellular Dermal Matrix to Treat Full Thickness Skin Defects: Follow-Up Subjective and Objective Skin Quality Assessments. Arch. Craniofacial Surg..

[40] Niu C., Aisa H.A. (2017). Upregulation of Melanogenesis and Tyrosinase Activity: Potential Agents for Vitiligo. Molecules.

[41] Callender V.D., St Surin-Lord S., Davis E.C., Maclin M. (2011). Postinflammatory hyperpigmentation: Etiologic and therapeutic considerations. Am. J. Clin. Dermatol..

[42] He T., Zhang Z., Zhang X., Niu H., Wang S., Wang Q., Lai C. (2025). Effects of Poly-L-Lactic Acid Fillers on Inflammatory Response and Collagen Synthesis in Different Animal Models. J. Cosmet. Dermatol..

[43] Bailly S., Ferrua B., Fay M., Gougerot-Pocidalo M.A. (1990). Differential regulation of IL 6, IL 1 A, IL 1 beta and TNF alpha production in LPS-stimulated human monocytes: Role of cyclic AMP. Cytokine.

[44] Carruthers J., Donofrio L., Hardas B., Murphy D.K., Jones D., Carruthers A., Sykes J.M., Creutz L., Marx A., Dill S. (2016). Development and Validation of a Photonumeric Scale for Evaluation of Facial Fine Lines. Dermatol. Surg..

