# Supplementary material for: Injectable Particulated Human Acellular Dermal Matrix Booster for Skin Restoration: An Integrated Randomized, Split-Face, Double-Blinded Clinical Trial and Preclinical Study
Source: Int J Mol Sci. 2026 Feb 26;27(5):2193. doi: 10.3390/ijms27052193 (PMC12985180; doi:10.3390/ijms27052193)
Supplement: Supplementary file 1 [file ijms-27-02193-s001.zip › ijms-4140971-supplementary.pdf]

Table S1. Allergan Cheek Smoothness Scale

**Score**	**Grade**	**Description**
0	None	Smooth visual skin texture
1	Minimal	Slightly coarse and uneven visual skin texture
2	Moderate	Moderately coarse and uneven visual skin texture: may have early elastosis
3	Severe	Severely coarse visual skin texture, crosshatched fine lines: may have some elastosis
4	Extreme	Extremely coarse visual skin texture, crosshatched deep creases: extreme elastosis

Table S2. Global Aesthetic Improvement Scale

Score	Description
1	Very much improved
2	Much improved
3	Improved
4	No change
5	Worse

Table S3. ELISA kit information

No.	Kit	Manufacturer	Cat.No
1	Human Pro-Collagen I α1 SimpleStep ELISA kit	Abcam	ab210966
2	Human Collagen TYPE III Alpha 1 ELISA kit	Novus Biologicals	NBP2-75858
3	Human Elastin ELISA Kit	MyBioSource	MBS704171
4	Human VEGF ELISA kit	Abcam	ab222510
5	Human FGF ELISA kit	Abcam	ab219636
6	Human PDGF BB ELISA Kit	Abcam	ab184860
7	Human TGFβ-1 ELISA kit	Abcam	ab100647
8	Hyaluronan Quantikine ELISA Kit	R&D Systems	DHYALO
9	Human Hyaluronan Synthase 2 ELISA Kit	MyBioSource	MBS28882985
10	Mouse IL-1β SimpleStep ELISA kit	Abcam	ab197742
11	Mouse IL-6 ELISA kit	R&D systems	M6000B-1
12	Mouse TNF-α SimpleStep ELISA kit	Abcam	ab208348
13	Mouse Prostaglandin E2 ELISA Kit	MyBioSource	MBS266212

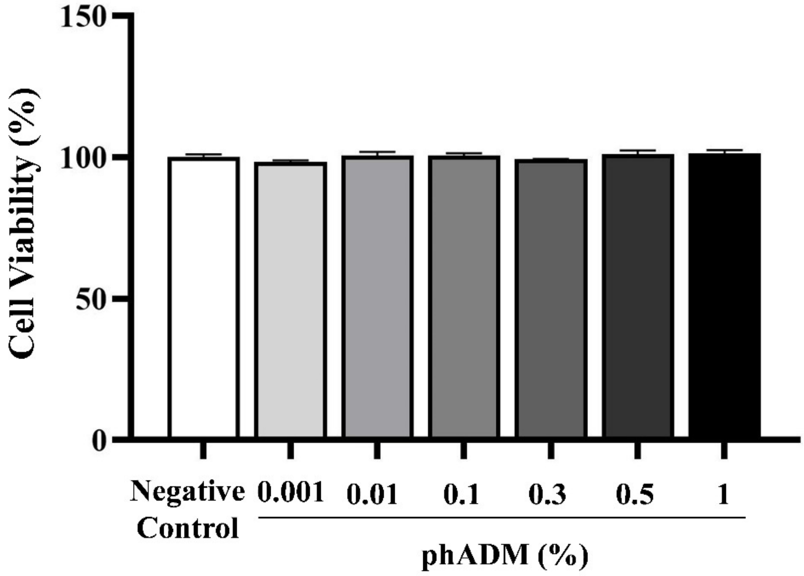

Figure S1. Cell viability of mouse macrophages after 24 hours of treatment with increasing concentrations of phADM (0.001 – 1%). Data are presented as mean ± SD. phADM, particulated human acellular dermal matrix.

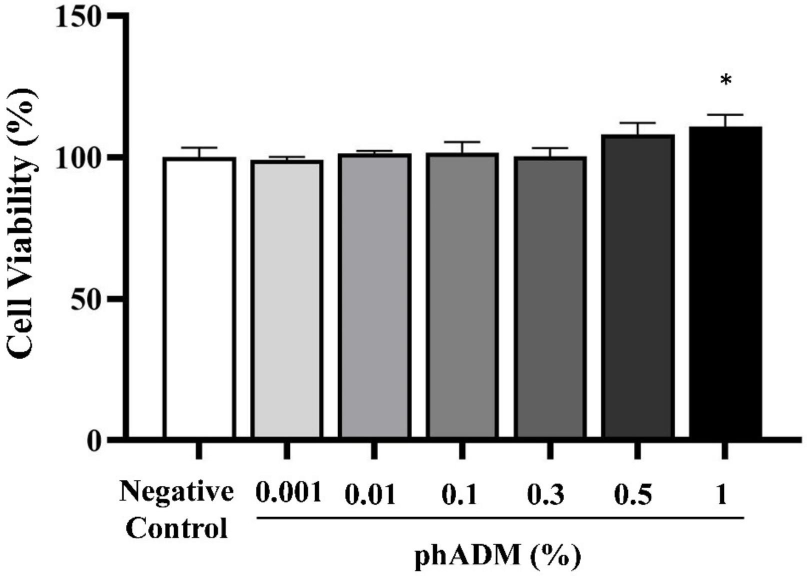

Figure S2. Cell viability of human epidermal keratinocytes after 24 hours of treatment with increasing concentrations of phADM (0.001 – 1%). Data are presented as mean ± SD. *comparison to negative control, *$p < 0.05$. phADM, particulated human acellular dermal matrix.

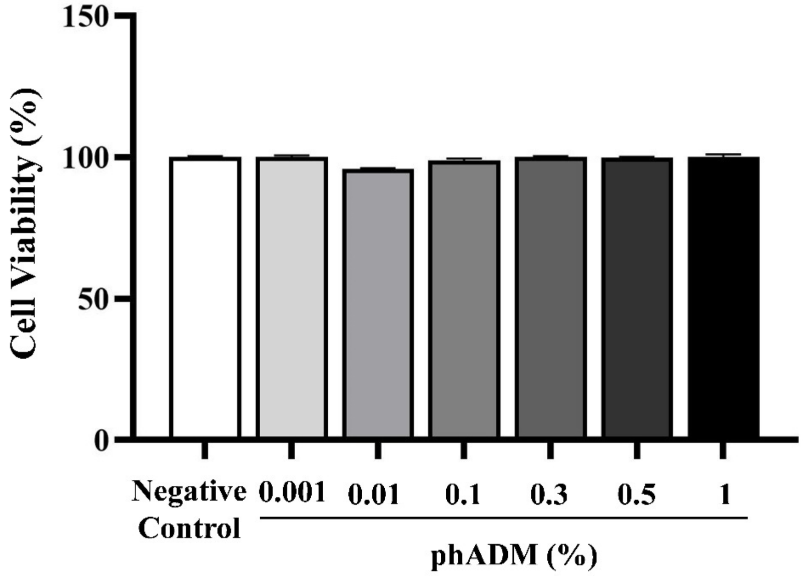

Figure S3. Cell viability of mouse melanoma cells after 72 hours of treatment with increasing concentrations of phADM (0.001 – 1%). Data are presented as mean ± SD. phADM, particulated human acellular dermal matrix.
